# Danhong injection attenuates doxorubicin-induced cardiotoxicity in rats *via* suppression of apoptosis: network pharmacology analysis and experimental validation

**DOI:** 10.3389/fphar.2022.929302

**Published:** 2022-08-22

**Authors:** Xiaojiao Yi, Fugen Wang, Yan Feng, Junfeng Zhu, Yongjiang Wu

**Affiliations:** ^1^ Department of Pharmacy, Affiliated Hangzhou Xixi Hospital, Zhejiang University School of Medicine, Hangzhou, China; ^2^ Department of Pharmacy, Cancer Hospital of the University of Chinese Academy of Sciences (Zhejiang Cancer Hospital), Institute of Basic Medicine and Cancer (IBMC), Chinese Academy of Sciences, Hangzhou, China; ^3^ College of Pharmaceutical Sciences, Zhejiang University, Hangzhou, China

**Keywords:** Danhong injection, doxorubicin, cardiotoxicity, network pharmacology, apoptosis

## Abstract

Doxorubicin (DOX) is a potent chemotherapeutic agent that is used against various types of human malignancies. However, the associated risk of cardiotoxicity has limited its clinical application. Danhong injection (DHI) is a Chinese medicine with multiple pharmacological activities and is widely used for treating cardiovascular diseases. The aim of the present study was to evaluate the potential protective effect of DHI on DOX-induced cardiotoxicity *in vivo* and to investigate the possible underlying mechanisms. First, a sensitive and reliable HPLC−ESI-Q-TOF-MS/MS method was developed to comprehensively analyze the chemical compositions of DHI. A total of 56 compounds were identified, including phenolic acids, tanshinones, and flavonoids. Then, a DOX-induced chronic cardiotoxicity rat model was established to assess the therapeutic effect of DHI. As a result, DHI administration prevented the reduction in body weight and heart weight, and improved electrocardiogram performance. Additionally, the elevated levels of serum biochemical indicators were reduced, and the activities of oxidative enzymes were restored in the DOX-DHI group. Network pharmacology analysis further revealed that these effects might be attributed to 14 active compounds (*e.g.*, danshensu, salvianolic acid A, salvianolic acid B, rosmarinic acid, and tanshinone IIA) and 15 potential targets (*e.g.*, CASP3, SOD1, NOS3, TNF, and TOP2A). The apoptosis pathway was highly enriched according to the KEGG analysis. Molecular docking verified the good binding affinities between the active compounds and the corresponding apoptosis targets. Finally, experimental validation demonstrated that DHI treatment significantly increased the Bcl-2 level and suppressed DOX-induced Bax and caspase-3 expression in rat heart tissue. Furthermore, DHI treatment obviously decreased the apoptosis rate of DOX-treated H9c2 cells. These results indicate that DHI attenuated DOX-induced cardiotoxicity via regulating the apoptosis pathway. The present study suggested that DHI is a promising agent for the prevention of DOX-induced cardiotoxicity.

## 1 Introduction

Doxorubicin (DOX) is a typical anthracycline antibiotic that was first extracted from *Streptomyces peucetius* in 1969 (Arcamone et al., 1969). It has a strong anti-tumor effect on a wide range of malignancies, such as lymphoma, pediatric leukemia, breast carcinoma, and osteosarcoma (Yu et al., 2018). However, clinical application of DOX is limited by severe and dose-dependent cardiotoxicity, which may cause irreversible congestive heart failure (CHF) (Singal et al., 1987; Singal and Iliskovic, 1998). In a retrospective study of 399 patients with advanced carcinoma, CHF occurred in about 4% of patients who had received a DOX dose of 501–550 mg/m^2^. The incidence rose to more than 18% at a dose of 551–600 mg/m^2^ and to about 36% when exceeding the dose of 601 mg/m^2^. Therefore, the total dose of DOX was suggested to be less than 550 mg/m^2^ to minimize the risk of serious cardiotoxicity (Lefrak et al., 1973).

The molecular pathogenesis of DOX-induced cardiotoxicity is still not entirely clear. Previous studies suggest that DOX-induced oxidative stress increases the production of free radicals (*e.g.*, reactive oxygen species (ROS)) (Wallace et al., 2020; Han et al., 2022). Under normal conditions, free radicals can be scavenged by antioxidant enzymes, such as superoxide dismutase (SOD) and glutathione peroxidase (GSH-Px). Meanwhile, DOX-derived ROS leads to an imbalance between oxidant generation and antioxidant defense, which results in free radical accumulation, lipid peroxidation, membrane disruption, and ultimately causes cell apoptosis (Doroshow, 1983; Mimnaugh et al., 1985; Childs et al., 2002). Thus, the apoptosis of cardiomyocytes is also involved in the development of DOX-induced cardiotoxicity and is one of the final consequences. It is well-documented that DOX induces apoptosis through the intrinsic mitochondrial pathway by modulating the expression of Bax, Bcl-2, and caspase-3 (Kluza et al., 2004; Abushouk et al., 2017), which subsequently causes mitochondrial damage, myofibrillar degeneration, and cardiac dysfunction. Because DOX remains the first-line agent for many cancer treatments, the search for safe and efficient remedies to attenuate DOX-induced myocardial damage remains urgent in clinical chemotherapeutic strategies.

Although enormous effort has been expended trying to prevent DOX-induced cardiotoxicity, there is little consensus on the best strategy. Many researches have reported that natural products and traditional Chinese medicine preparations (TCMPs) have unique advantages in reducing DOX-induced heart damage (Abushouk et al., 2017; Zhu et al., 2021). Danhong injection (DHI) is a modern Chinese medical product that is prepared from *Salvia miltiorrhiza* Bunge [Lamiaceae] (Danshen) and *Carthamus tinctorius* L. [Asteraceae] (Honghua). DHI has multiple pharmacological properties, including anti-oxidant, anti-apoptotic, anti-inflammatory, anti-coagulation, and pro-angiogenesis, and is widely applied in cardio- and cerebro-vascular diseases (Feng et al., 2019; Wang et al., 2021). In our previous study, the protective effect of DHI on DOX-induced cardiotoxicity was preliminarily confirmed in H9c2 cells (Yi et al., 2018). However, its effect and mechanisms have not yet been studied in DOX-induced cardiotoxic animals. Consequently, the present study was designed to assess the therapeutic effect of DHI on DOX-induced myocardial damage in rats and to elucidate its possible molecular mechanisms.

Network pharmacology, which is based on chemoinformatics, bioinformatics, network biology, and network analysis, has already become a powerful tool to elucidate the mechanisms of traditional Chinese medicines (TCMs). It systematically studies the multilevel interactions among components, targets, and diseases, which is in accordance with the holistic theory of TCMs (Zhu et al., 2019; An et al., 2020; Yang et al., 2021; Chen et al., 2022). Therefore, the present study employed network pharmacology combined with molecular docking verification and experimental validation to clarify the possible mechanisms of DHI against DOX-induced cardiotoxicity. The findings may lay a foundation for the clinical use of DHI in attenuating DOX-induced myocardial damage.

## 2 Materials and methods

### 2.1 Chemicals and reagents

DHI was obtained from Shandong Danhong Pharmaceutical Co., Ltd. (Heze, Shandong, China). DOX was produced by Shenzhen Main Luck Pharmaceuticals Inc. (Shenzhen, Guangdong, China) and dissolved in normal saline before the experiment. Commercially available standards (uridine, 5-hydroxymethylfurfural, danshensu, protocatechuic acid, protocatechuic aldehyde, p-coumaric acid, salvianolic acid D, rosmarinic acid, lithospermic acid, salvianolic acid B, and salvianolic acid A) were obtained from Chengdu MUST Biotechnology Co., Ltd. (Chengdu, Sichuan, China) for compound identification (purity > 98%). HPLC-grade acetonitrile and formic acid were purchased from J&K Scientific Ltd. (Shanghai, China). Cell culture reagents, including Dulbecco’s modified Eagle’s medium (DMEM) and fetal bovine serum (FBS), were produced by Gibco BRL (Calsbad, CA, United States). AnnexinV-FITC/PI apoptosis detection kit was obtained from MultiSciences (Lianke) Biotech Co., Ltd. (Hangzhou, Zhejiang, China). Malondialdehyde (MDA, #A003-1), SOD (#A001-1), and GSH-Px (#A005) assay kits were purchased from Nanjing Jiancheng Bioengineering Institute (Nanjing, Jiangsu, China). Antibodies specific for Bax (#ab32503), Bcl-2 (#ab196495), and caspase-3 (#ab184787) were obtained from Abcam (Cambridge, United Kingdom), while GAPDH (#AF0006) was obtained from Beyotime Biotechnology (Shanghai, China). Deionized water was prepared using a Milli-Q ultrapure water system (Millipore, Milford, MA, United States). All of the other reagents and chemicals that were used in this study were of analytical grade.

### 2.2 Profiling the chemical constituents in Danhong injection

The chemical profiling of DHI was performed using an UPLC system (Waters Technologies Co., Ltd., Milford, MA, United States) coupled with a Triple TOF 5600 plus mass spectrometer (AB SCIEX, Framingham, MA, United States). DHI was used directly and the injection volume was 5 μL. The chromatographic separation was carried out on an Agilent Zorbax SB-C18 column (4.6 mm × 250 mm, 5 μm; Agilent Technologies, Santa Clara, CA, United States) at a flow rate of 1.0 ml/min at 35°C. The optimal mobile phase consisted of 0.5% formic acid in water (A) and acetonitrile (B) with a gradient elution program, as follows: 0–15 min, 2–10% B; 15–20 min, 10–17% B; 20–45 min, 17–28% B; 45–48 min, 28–60% B; and 48–53 min, 60% B.

The MS detection was conducted in both positive (ESI+) and negative (ESI−) ion modes with the following optimized parameters: full scan range, *m/z* 50–1,500; spray voltage, +5.5 kV (ESI+) and −4.5 kV (ESI−); source temperature, 600°C (ESI+) and 550°C (ESI−); Gas 1 and Gas 2 (Air), 50 psi; CUR (N_2_), 30 psi; and collision voltage, 40 ± 20 eV. Dynamic background subtract (DBS) information-dependent acquisition (IDA) was used to trigger the acquisition of MS/MS spectra for low concentration level constituents. Meanwhile, the automated calibration delivery system (CDS) was employed for exact mass calibration before each analysis. The accurate mass and composition of the precursor and fragment ions were processed using the PeakView software (version 1.2.0.3; AB SCIEX) that was supplied with the instrument.

### 2.3 Animal experiment

#### 2.3.1 Animals

Male Sprague–Dawley rats (200 ± 20 g, *n* = 40) were purchased from Zhejiang Experimental Animal Center (Hangzhou, Zhejiang, China) and acclimated in the laboratory for 1 week before the experiment. Rats were kept under standard conditions of temperature (20 ± 2°C) and humidity (50 ± 10%) in a 12-h light/dark cycle, with food and water provided *ad libitum*. The animal experiments were carried out in accordance with the Guide for the Care and Use of Laboratory Animals. The protocol was approved by the Animal Ethic Review Committees of Zhejiang Cancer Hospital.

#### 2.3.2 Experimental design

**FIGURE 1:**
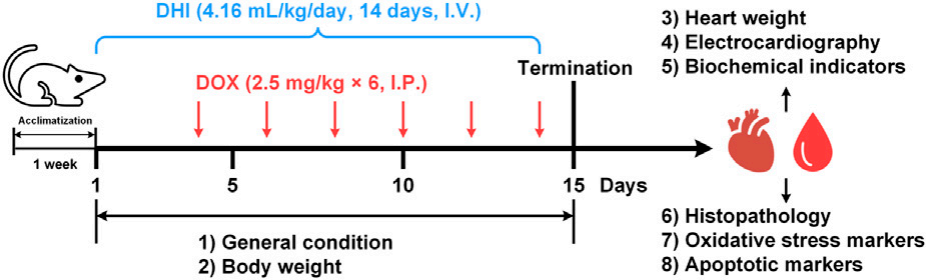
Schematic diagram of the animal experiment design. DHI: Danhong injection; DOX: doxorubicin; I.V.: intravenously; I.P.: intraperitoneal.

The rats were randomly divided into four groups and treated as follows (Figure 1):

1) CON group (*n* = 8): Normal control rats, received only normal saline for 14 consecutive days.
2) DOX group (*n* = 12): DOX-intoxicated rats, received 2.5 mg/kg DOX intraperitoneally every other day for a total of six injections. The cumulative dose of DOX was 15 mg/kg as previously described (Chen et al., 2014).
3) DHI group (*n* = 8): DHI only-treated rats, received an intravenous injection of 4.16 ml/kg DHI for 14 consecutive days. The DHI dose was determined based on the clinical daily dose (40 ml DHI/60 kg person) according to the dose normalization by body surface area (Reagan-Shaw et al., 2008; Li et al., 2016).
4) DOX–DHI group (*n* = 12): DOX and DHI co-treated rats, received 4.16 ml/kg DHI intravenously for 14 consecutive days and 2.5 mg/kg DOX intraperitoneally on 4th, 6th, 8th, 10th, 12th, and 14th days.

The general condition and body weight of the rats were recorded daily throughout the experimental period. After 24 h of the last DOX administration (15th day), all rats were anesthetized with sodium pentobarbital and subjected to electrocardiogram (ECG) monitoring. The rats were then sacrificed and blood samples were collected from the abdominal aorta for biomedical analysis. Heart tissues were dissected out for histopathological examination, oxidative stress markers measurement, and western blot analysis.

#### 2.3.3 Electrocardiography

ECG was recorded using the SP2006 ECG analysis system (Softron, Beijing, China). Anesthetized rats were placed on the operating table in supine position and needle electrodes were inserted subcutaneously into the four limbs at position II (right forelimb to left hindlimb). The rate and rhythm disorders were evaluated by measuring the changes in the ECG parameters.

#### 2.3.4 Detection of biochemical indicators in serum

For serum preparation, blood samples were kept at room temperature for 30 min to allow them to clot. After centrifugation at 5,000 rpm for 5 min at 4°C, the supernatants were collected and used for detection of serum cardiotoxicity indices, such as alanine aminotransferase (ALT), aspartate aminotransferase (AST), lactate dehydrogenase (LDH), creatine kinase (CK), and creatine kinase isoenzyme (CK-MB), using an automatic biochemical blood analyzer (Model 7180; Hitachi, Tokyo, Japan).

#### 2.3.5 Histopathological examination

Heart specimens were fixed in 10% neutral formalin for 24 h, dehydrated in graded ethanol, cleared with xylene, and processed to prepare 5-µm paraffin sections. Sections were stained with hematoxylin and eosin and examined under a light microscope at ×100 magnification (DM4000; Leica Microsystems, Wetzlar, Germany).

#### 2.3.6 Measurement of oxidative stress markers in cardiac tissue

Frozen heart tissues were homogenized in nine volumes of ice-cold saline to obtain 10% (w/v) homogenate (Model PRO 200 homogenizer; PRO Scientific, Monroe, CT, United States). After centrifugation at 5,000 rpm for 5 min at 4°C, the supernatants were collected and used for measurement of oxidative stress markers activities, such as MDA, SOD, and GSH-Px, according to the manufacturer’s instructions for the available commercial kits.

### 2.4 Network pharmacology analysis

#### 2.4.1 Identification of candidate active components in Danhong injection

Based on the results of chemical profiling of DHI, the identified compounds were regarded as candidate components and then screened according to an *in silico* ADME model provided by Traditional Chinese Medicine Systems Pharmacology Database and Analysis Platform (TCMSP, https://old.tcmsp-e.com/tcmsp.php). The inclusion criterion of components was drug-likeness (DL) ≥ 0.18. In addition, the compounds with low DL value but possessed high contents and significant pharmacological effects were also adopted as candidate active components.

#### 2.4.2 Collection of targets of candidate active components

The related targets of candidate active components were obtained from TCMSP and SwissTargetPrediction (http://www.swisstargetprediction.ch/). In TCMSP, the compound name was used as the keyword for target collection. In addition, the canonical SMILES information of the candidate active components was obtained from PubChem (https://pubchem.ncbi.nlm.nih.gov/) and was imported into the SwissTargetPrediction to predict the potential targets of compounds. Only the targets with probability >0.5 were retained. All of the targets were further normalized to official symbols using the Uniprot Knowledgebase (http://www.uniprot.org/) with the species restricted to “*Homo sapiens*.”

#### 2.4.3 Collection of targets related to doxorubicin-induced cardiotoxicity

The targets related to DOX-induced cardiotoxicity were obtained from the Pharmacogenomics Knowledgebase (PharmGKB, https://www.pharmgkb.org/) and the Human Gene Database (GeneCards, https://www.genecards.org/). In particular, the genes in the “doxorubicin pathway of cardiomyocyte cell” were selected in PharmGKB, while in GeneCards, the targets were derived using exact search phrases, such as “doxorubicin-induced cardiotoxicity,” “adriamycin-induced heart failure,” and “doxorubicin-induced cardiomyopathy.” After removing the duplicate targets, the remaining targets were standardized using the Uniprot database.

#### 2.4.4 Construction of active compound–potential target network

The targets related to DHI and DOX-induced cardiotoxicity were imported to the online tool Draw Venn Diagram (http://bioinformatics.psb.ugent.be/webtools/Venn/) for intersection analysis. The overlapping targets were screened as the potential targets of DHI against DOX-induced cardiotoxicity. After removing any candidate active components that cannot act on the potential targets, the remaining components and their corresponding potential targets were introduced into the Cytoscape software (version 3.8.2) to construct the active compound-potential target network.

#### 2.4.5 Enrichment pathway analysis

Kyoto Encyclopedia of Genes and Genomes (KEGG) enrichment analysis of the potential targets was performed using the clusterProfiler package in R software (version 4.1.0) to identify the key pathways that played significant roles in DHI against DOX-induced cardiotoxicity. Pathways with adjusted *p* < 0.05 were considered to be statistically significant.

### 2.5 Molecular docking verification

Molecular docking was employed to verify the results of network pharmacology using the AutoDockTools software (version 1.5.6; http://mgltools.scripps.edu/). Briefly, the crystal structures of the targets were retrieved from the RCSB PDB database (https://www.rcsb.org/). The obtained receptors were embellished using the PyMOL software (version 2.5.0; Schrödinger, LLC, New York, NY, United States) to delete original ligands, solvent molecules, and organic molecules. Meanwhile, the three-dimensional structures of the compounds were downloaded in sdf format from the PubChem database (https://pubchem.ncbi.nlm.nih.gov/). The obtained ligands were imported into the ChemBio3D Ultra software (version 12.0; CambridgeSoft Corp., Waltham, MA, United States) for energy minimization by MM2 force field optimization (Li et al., 2021). The receptors and ligands were further prepared by AutoDockTools, including adding hydrogens, detecting rotatable bonds, and setting docking parameters. Finally, the saved pdbqt files were used for molecular modeling and the docking score was calculated to evaluate the binding capacity of the compound–target interaction. The conformation with the lowest binding energy was considered as the most suitable binding model and was visualized using PyMOL.

### 2.6 Experimental validation

#### 2.6.1 Assessment of apoptotic markers in rat heart tissue

The expression of apoptotic markers (Bax, Bcl-2, and caspase-3) in rat heart tissue were determined by western blot analysis. The total protein of heart tissues was extracted by complete homogenization in 500 μl of RIPA lysis buffer and subsequent centrifugation at 11,000 rpm for 20 min at 4°C. The supernatants were collected and protein concentrations were measured using the bicinchoninic acid assay. Equal amounts of samples (20 μg) were electrophoresed using 12% SDS-PAGE and then transferred onto the PVDF membranes using a Mini-PROTEAN Tetra System (Bio-Rad, Hercules, CA, United States). Next, the membranes were blocked with 5% bovine serum albumin for 1 h at room temperature and then incubated at 4°C overnight with specific primary antibodies (Bax, Bcl-2, caspase-3, and GAPDH). After washing with tris-buffered saline with Tween solution (TBST), the membranes were incubated with secondary antibodies (goat anti-rabbit IgG or goat anti-mouse IgG) for 2 h at room temperature. The proteins on the blot were visualized using chemiluminescence. The density of each band was normalized to GAPDH and protein expression was calculated using the ImageJ software (NIH, Bethesda, MD, United States).

#### 2.6.2 Detection of apoptosis in H9c2 cells

H9c2 cells derived from rat embryonic myocardium were provided by the Cell Bank of Chinese Academy of Sciences (Shanghai, China). Cells were cultured in DMEM containing 10% FBS at 37°C in a humidified atmosphere of 95% air and 5% CO_2_. Cells in the logarithmic growth phase were seeded into a six-well plate at a density of 1 × 10^5^/well and divided into four groups (*n* = 3): CON group, no treatment; DOX group, 0.9 μM DOX; DHI group, 10 μL/ml DHI; and DOX–DHI group, 0.9 μM DOX and 10 μL/ml DHI. The doses of DOX and DHI were determined based on our previous study (Yi et al., 2018).

The cell apoptosis was detected by flow cytometry assay according to the protocols of the apoptosis detection kit. After 48 h of treatment, cells were digested using pancreatic enzymes without EDTA and collected by centrifugation at 2,000 rpm. The harvested cells were washed twice with cold PBS and re-suspended in 100 μl binding buffer. Then, 5 μl Annexin V-FITC and 10 μl PI were added to the suspension in the dark for 5 min. After washing with PBS, cells were analyzed using an Accuri C6 flow cytometer (BD Biosciences, San Jose, CA, United States).

### 2.7 Statistical analysis

Data were presented as mean ± standard error of the mean (SEM). GraphPad Prism software (version 6.0; La Jolla, CA, United States) was used for statistical analysis. The level of significance between groups was determined using one-way analysis of variance (ANOVA) followed by Fisher’s LSD multiple comparison. In all cases, *p* < 0.05 was considered to be statistically significant.

## 3 Results

### 3.1 Identification of chemical constituents in Danhong injection

**FIGURE 2:**
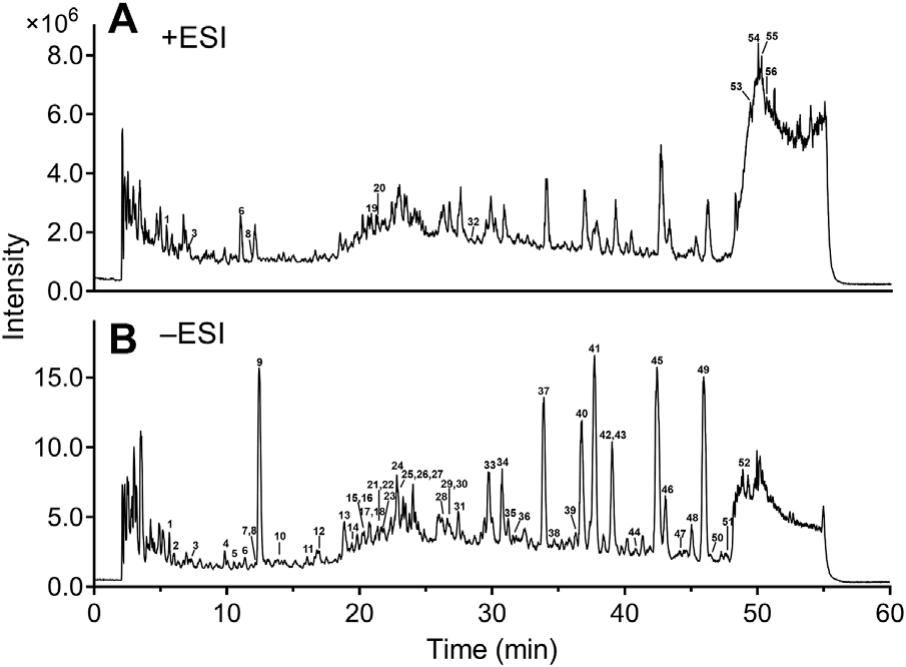
Total ion chromatograms of DHI in positive **(A)** and negative **(B)** ion modes. DHI: Danhong injection; ESI: electrospray ionization. Column: Agilent Zorbax SB-C18 (4.6 mm × 250 mm, 5 μm); mobile phase A: 0.5% formic acid in water; mobile phase B: acetonitrile; solvent gradient: 0–15 min, 2–10% B; 15–20 min, 10–17% B; 20–45 min, 17–28% B; 45–48 min, 28–60% B; 48–53 min, 60% B; flow rate: 1.0 ml/min; temperature: 35°C; injection volume: 5 μl.

Our previous studies have been conducted on the main constituents and content of DHI (Liu et al., 2013; Zhu et al., 2017). In the present study, a sensitive and reliable HPLC−ESI-Q-TOF-MS/MS method was developed for comprehensively identification of the chemical compositions in DHI. The total ion chromatograms (TIC) of DHI in positive and negative ion modes are displayed in Figure 2. LC−MS/MS analysis revealed 56 compounds in DHI belonging to phenolic acids, tanshinones, flavonoids, nucleosides, terpenes, and others (Supplementary Table S1). Meanwhile, 11 compounds (compound 1, 5, 9, 10, 13, 31, 37, 41, 43, 45, and 49) were unambiguously identified by comparing with the reference standards. For other compounds, the structures were tentatively assigned based on their retention time, exact molecular weight, and MS/MS spectra by referring to the chemical information with those in the literature (Zhang et al., 2016; Zhu et al., 2017; Li et al., 2019) and databases, such as Reaxys (https://www.reaxys.com/), PubMed (https://pubmed.ncbi.nlm.nih.gov/), and CNKI (https://www.cnki.net/). The chemical structures of these compounds are presented in Supplementary Figure S1.

### 3.2 Animal experiment

#### 3.2.1 Effect of Danhong injection on general condition of rats

The diet and general activity of rats were observed every day during the entire experimental process. In the first 3 days, rats in each group were in good mental state, maintained normal feeding and drinking habits, and had smooth and shiny hair. On day 4, the rats in the DOX and DOX–DHI groups began to exhibit signs of low spirit, decreased food intake, slow movement, and lusterless hair. They also experienced eye hyperemia and loose stool at the end of the experiment. The symptoms in the DOX–DHI group were less severe and appeared later than those in the DOX group. In the control and DHI groups, the rats were in a good general condition during the whole experimental period and no abnormalities were observed.

#### 3.2.2 Effect of Danhong injection on body weight and heart weight

**FIGURE 3:**
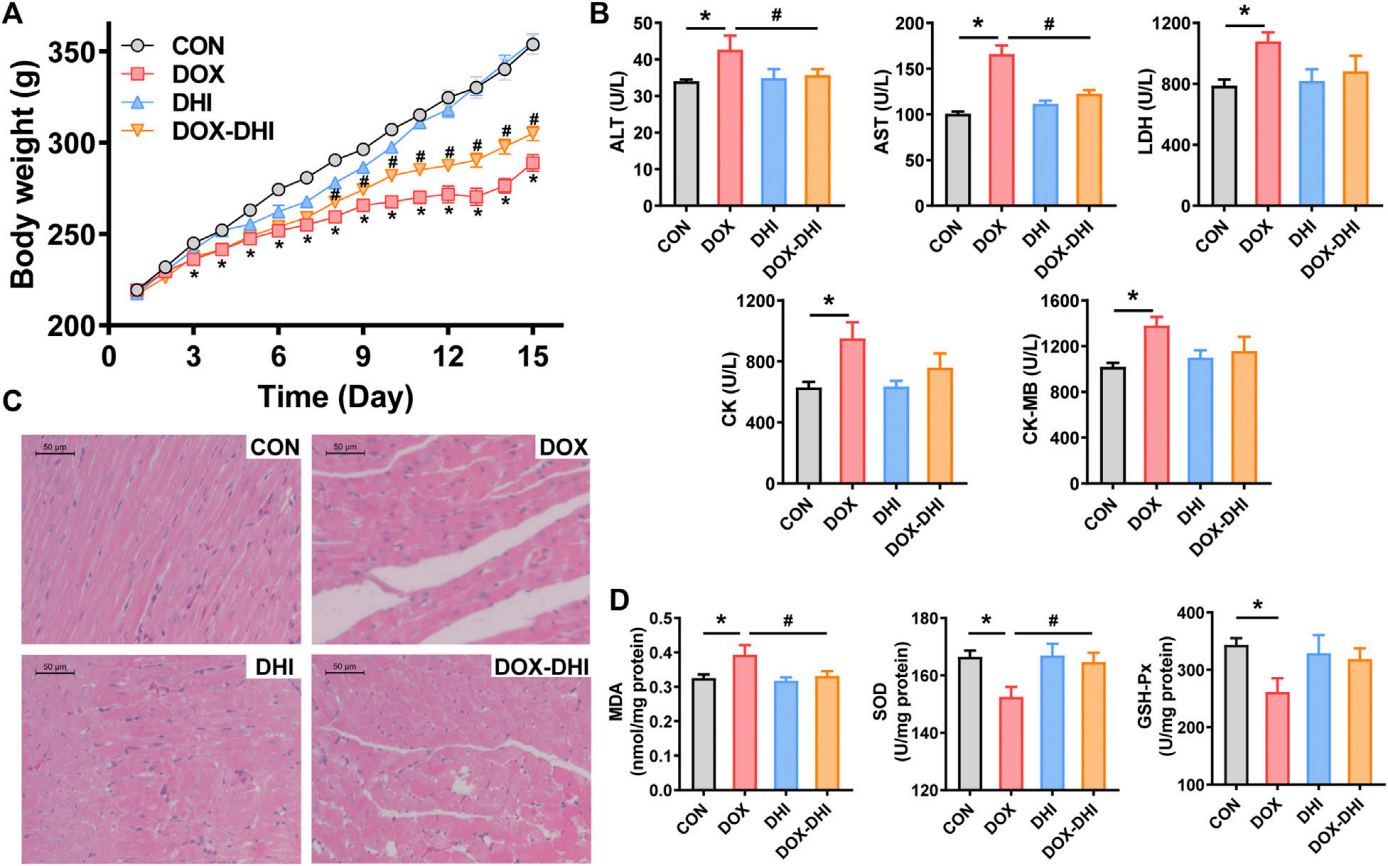
Protective effect of DHI on DOX-induced cardiotoxicity in rats. **(A)** Body weight changes of rats in each group. **(B)** Detection of serum biochemical markers (ALT, AST, LDH, CK, and CK-MB). **(C)** Histological alterations in heart tissue (10 × 10). **(D)** Measurement of oxidative stress markers (MDA, SOD, and GSH-Px) in heart tissue. CON: normal control; DHI: Danhong injection; DOX: doxorubicin; ALT: alanine aminotransferase; AST: aspartate aminotransferase; LDH: lactate dehydrogenase; CK: creatine kinase; CK-MB: creatine kinase isoenzyme; MDA: malondialdehyde; SOD: superoxide dismutase; GSH-Px: glutathione peroxidase. Data are expressed as mean ± SEM (CON and DHI, *n* = 8; DOX and DOX–DHI, *n* = 12). ^*^ *p* < 0.05 versus the CON group. ^#^ *p* < 0.05 versus the DOX group.

After the beginning of the experiment, the rats were weighed daily to record the changes in body weight (Figure 3A and Table 1). The initial body weight (Initial BW) was similar among all of the groups (*p* > 0.05). The weight increased steadily in each group during the experimental period. The final body weight (Final BW) of the rats was recorded at the end of the experiment and BW gain was calculated according to the following formula: BW gain = (Final BW—Initial BW)/Initial BW × 100. Rats in the control and DHI groups had the most significant BW gain, whereas BW gain in the DOX group was the lowest and the difference was significant when compared to the CON group (*p* < 0.05). Furthermore, the treatment with DHI in DOX-intoxicated rats partially prevented a decrease in BW gain when compared to the DOX group (*p* < 0.05).

**TABLE 1:** Effect of DHI on DOX-induced changes in BW, HW, and HI.

Group	CON	DOX	DHI	DOX–DHI
Initial BW (g)	219.25 ± 1.99	219.33 ± 1.45	217.00 ± 2.34	215.40 ± 2.26
Final BW (g)	353.75 ± 5.46	288.90 ± 4.60^*^	355.25 ± 4.12	305.00 ± 3.90^#^
BW gain (%)	61.52 ± 3.45	33.44 ± 2.40^*^	59.89 ± 3.55	42.59 ± 1.87^#^
HW (g)	1.49 ± 0.019	1.01 ± 0.002^*^	1.52 ± 0.016	1.08 ± 0.018^#^
HI (%)	0.42 ± 0.002	0.35 ± 0.004^*^	0.43 ± 0.003	0.37 ± 0.005^#^

CON: normal control; DHI: Danhong injection; DOX: doxorubicin; BW: body weight; HW: heart weight; HI: heart index. Data are expressed as mean ± SEM (CON and DHI, *n* = 8; DOX and DOX–DHI, *n* = 12). ^*^ *p* < 0.05 versus the CON group; ^#^ *p* < 0.05 versus the DOX group.

After the rats were anesthetized, the hearts were quickly removed, washed with precooled saline, and gently wiped using filter paper. The wet heart weight (HW) was recorded and the heart index (HI) was calculated according to the following formula: HI = (HW/Final BW) × 100. The HW and HI in the DOX group were significantly lower than those in the control group (*p* < 0.05; Table 1). These changes were alleviated by the DHI treatment (*p* < 0.05). These results indicated that the administration of DHI somewhat prevented the reduction in BW, HW, and HI induced by DOX.

#### 3.2.3 Effect of Danhong injection on electrocardiography

The ECG showed a normal pattern in the control and DHI groups (Table 2). The heart rate (HR) in the DOX group was observably lower than that in the CON group (*p* < 0.05). The DHI treatment significantly increased the HR of rats (*p* < 0.05). Compared to the CON group, the rats in the DOX group showed several ECG changes, including deepening S-wave, high amplitude T-wave, and prolongation of PR, QT, and QTc intervals. These ECG abnormalities were visibly improved in DOX-intoxicated rats treated with DHI (*p* < 0.05). The QRS complex showed no differences among all of the groups.

**TABLE 2:** ECG parameters of rats treated with DHI and/or DOX.

Group	CON	DOX	DHI	DOX–DHI
Heart rate (beats/min)	362.2 ± 15.2	300.9 ± 7.2^*^	357.6 ± 5.1	345.9 ± 11.8^#^
S wave (mv)	−60.3 ± 1.5	−86.7 ± 5.8^*^	−64.5 ± 4.8	−66.1 ± 2.7^#^
T wave (mv)	16.5 ± 0.7	28.0 ± 1.8^*^	17.4 ± 1.9	19.0 ± 0.8^#^
QRS (ms)	18.7 ± 0.8	20.2 ± 0.7	18.8 ± 0.8	19.8 ± 0.8
QT (ms)	64.2 ± 3.5	81.7 ± 1.4^*^	66.1 ± 1.6	72.5 ± 1.0^#^
PR (ms)	43.7 ± 2.0	57.1 ± 0.9^*^	45.0 ± 0.7	49.0 ± 0.7^#^
QTc (ms)	184.1 ± 4.9	220.6 ± 2.1^*^	186.8 ± 4.3	190.2 ± 3.6^#^

ECG: electrocardiogram; CON: normal control; DHI: Danhong injection; DOX: doxorubicin. Data are expressed as mean ± SEM (CON and DHI, *n* = 8; DOX and DOX-DHI, *n* = 12). ^*^ *p* < 0.05 versus the CON group; ^#^ *p* < 0.05 versus the DOX group.

#### 3.2.4 Effect of Danhong injection on serum biochemical indicators

The myocardial injury markers in serum were detected using an automatic biochemical analyzer (Figure 3B). Serum levels of ALT, AST, LDH, CK, and CK-MB in the DOX group were significantly increased when compared to the control group (*p* < 0.05). Treatment with DHI in DOX-intoxicated rats reduced serum ALT and AST levels in comparison to the DOX group (*p* < 0.05). In addition, the elevated levels of LDH, CK, and CK-MB induced by DOX were also reduced by DHI, although the differences were not significant. Moreover, there was no significant change in serum levels of ALT, AST, LDH, CK, and CK-MB between the control and DHI groups (*p* > 0.05).

#### 3.2.5 Effect of Danhong injection on cardiac histopathology

To further evaluate the effect of DHI on DOX-induced cardiotoxicity, histopathological examination of heart tissues was conducted at the end of the experiment. Hearts from the CON group showed intact cell structure, neat myocardial fiber arrangement, and no obvious histopathological changes (Figure 3C). Meanwhile, DOX administration led to disordered cardiac fibers, extensive cytoplasmic vacuolation, and severe myofiber degeneration. Relatively slighter changes were observed in the DOX–DHI group, indicating that the DHI treatment somewhat improved the cardiac structure of DOX-intoxicated rats. In addition, DHI may also have some effect on the histomorphological structure of the myocardial tissues.

#### 3.2.6 Effect of Danhong injection on cardiac oxidative stress markers

MDA is the final product of lipid peroxidation and is often used as a biomarker of oxidative damage in the heart. Induction of cardiotoxicity with DOX significantly increased MDA level compared to the control group (*p* < 0.05; Figure 3D). At the same time, DHI treatment in DOX-intoxicated rats decreased the MDA level in comparison to the DOX group (*p* < 0.05). Statistical analysis also showed that there was no significant difference in the MDA level between the control and DHI groups.

SOD is an important antioxidant enzyme that can protect the cells from oxidative damage. The results of the present study revealed that the SOD level in rat myocardia was significantly decreased in the presence of DOX when compared to the control group (*p* < 0.05; Figure 3D), whereas animals receiving both DOX and DHI had a higher SOD activity than those in the DOX group (*p* < 0.05). Moreover, no statistically significant difference was found in the SOD level between the control and DHI groups.

GSH-Px is also one of the key enzymes of the antioxidant system that can provide defense against hydrogen peroxide-mediated oxidative injury. The GSH-Px levels in DOX-exposed animals were reduced compared to those in the CON group (*p* < 0.05; Figure 3D). This trend was eliminated after DHI treatment in DOX-intoxicated rats, although this effect was not statistically significant. The GSH-Px level was not significantly different between the control and DHI groups.

### 3.3 Network pharmacology analysis

#### 3.3.1 Candidate active components in Danhong injection

A comprehensive analysis of the complex chemical compositions of TCMPs is conducive to illustrate the pharmacological mechanisms. In the present study, the 56 compounds identified by high-resolution mass spectrometry were selected as candidate components to ensure the accuracy and objectivity of the research. Moreover, only the compounds with good pharmacodynamics and pharmacokinetic properties can exert therapeutic effects. DL is an important parameter for evaluating whether a compound can be developed as a potential drug (Pak et al., 2022). Finally, 40 candidate active components were filtered out through ADME screening. Detailed information of these components is given in Supplementary Table S2.

#### 3.3.2 Target collection and construction of active compound–potential target network

**FIGURE 4:**
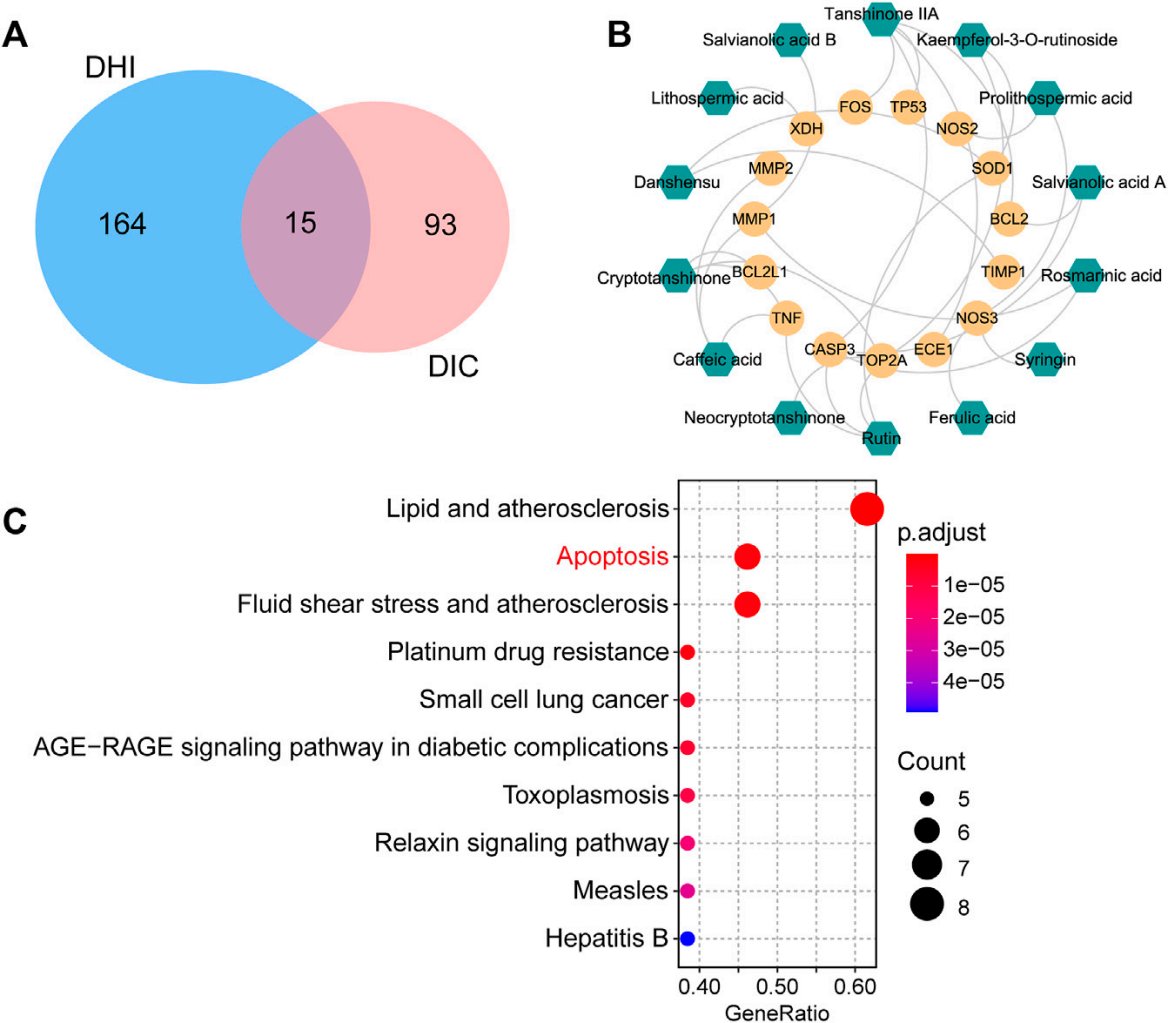
Network pharmacology analysis for clarifying the possible mechanisms of DHI against DOX-induced cardiotoxicity. **(A)** Venn diagram of common targets between DHI and DIC. **(B)** Active compound–potential target network. The green hexagons represent active compounds and the yellow circles represent potential targets. **(C)** Top 10 enriched KEGG pathways of the potential targets. DHI: Danhong injection; DIC: DOX-induced cardiotoxicity.

A total of 179 targets of candidate active components were obtained from TCMSP and SwissTargetPrediction databases, of which nine compounds failed for target collection. A total of 108 targets related to DOX-induced cardiotoxicity were derived from PharmGKB and GeneCards databases. Intersection analysis found that 15 targets related to DOX-induced cardiotoxicity were targeted by the compounds of DHI, which were considered as the potential targets of DHI against DOX-induced cardiotoxicity (Figure 4A). Detailed information of these potential targets is given in Supplementary Table S3. A TCMP–compound–target–disease network was established, which contained 31 candidate active components, 179 compound targets, and 108 disease targets (Supplementary Figure S2). After removing 26 candidate active components that cannot act on the potential targets, 14 active compounds remained. Then, an active compound–potential target network consisting of 29 nodes and 30 edges was constructed by Cytoscape (Figure 4B). Of these, the green hexagons represented active compounds (*e.g.*, danshensu, salvianolic acid A, salvianolic acid B, rosmarinic acid, and tanshinone IIA) and the yellow circles represented potential targets (*e.g.*, CASP3, SOD1, NOS3, TNF, and TOP2A), indicating that these compounds and targets were the potential pharmacodynamic substances and targets of DHI against DOX-induced cardiotoxicity.

#### 3.3.3 Enrichment pathway analysis

To investigate the underlying mechanisms of DHI against DOX-induced cardiotoxicity, enrichment pathway analysis was performed and a total of 73 KEGG pathways were significantly enriched. The top 10 key pathways are presented in Figure 4C, including lipid and atherosclerosis, apoptosis, and AGE-RAGE signaling pathway in diabetic complications. The correlation between these pathways and DOX-induced cardiotoxicity was investigated through text mining. It was found that apoptosis played a major role in the development of DOX-induced cardiotoxicity, while other highly enriched pathways (*e.g.*, lipid and atherosclerosis, fluid shear stress and atherosclerosis) showed no strong association with DOX-induced cardiotoxicity. Therefore, we speculated that the therapeutic effect of DHI was probably associated with the apoptosis pathway.

### 3.4 Molecular docking verification

**FIGURE 5:**
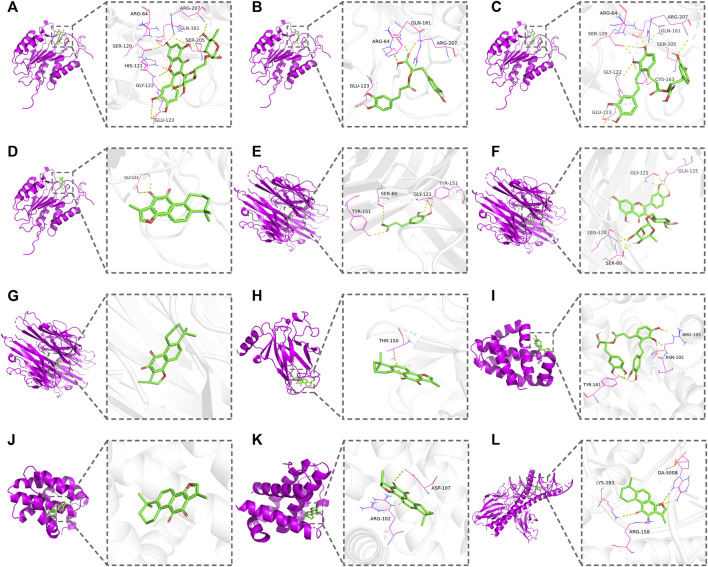
Docking patterns of the active compounds and the corresponding apoptosis pathway-related targets. **(A)** CASP3–rutin. **(B)** CASP3–rosmarinic acid. **(C)** CASP3–salvianolic acid A. **(D)** CASP3–tanshinone IIA. **(E)** TNF–caffeic acid. **(F)** TNF–rutin. **(G)** TNF–cryptotanshinone. **(H)** TP53–tanshinone IIA. **(I)** BCL2–salvianolic acid A. **(J)** BCL2–tanshinone IIA. **(K)** BCL2L1–cryptotanshinone. **(L)** FOS–tanshinone IIA.

The AutoDockTools software was used for molecular docking to prove the reliability of the findings obtained by network pharmacology. Based on the results of KEGG pathway analysis, six targets in the apoptosis pathway (CASP3, BCL2, TP53, TNF, BCL2L1, and FOS) were selected as the binding receptors. Meanwhile, the active compounds that interacted with these targets were derived from the active compound–potential target network and regarded as the docking ligands, including rutin, rosmarinic acid, salvianolic acid A, tanshinone IIA, caffeic acid, and cryptotanshinone. The binding domains of the receptors were determined according to the binding sites of the original ligands or the published studies (Anuradha et al., 2019; Liu et al., 2021; Tu et al., 2021; Verma et al., 2021). Docking parameters and the details of compounds and targets are presented in Table 3. The binding score was calculated to evaluate the binding affinity of the compound–target interaction. As shown in Table 3, the binding energies of the docking results were in the range of −8.73 to −4.93 kcal/mol. The lowest binding energy was observed in the TP53–tanshinone IIA complex, and caffeic acid showed the highest docking score against TNF. These results indicate that the active compounds in DHI had stable binding with the corresponding apoptosis pathway-related targets, and further validate that DHI exerted the protective effect on DOX-induced cardiotoxicity through the apoptosis pathway. The three-dimensional conformation of these complexes with the lowest binding energy is shown in Figure 5.

**TABLE 3:** Docking parameters and results of the active compounds and the corresponding apoptosis pathway-related targets.

Target name	PDB id	Center coordinates (x, y, z)	Size (x, y, z)	Compound name	PubChem CID	Binding energy (kcal/mol)
CASP3	1GFW	36.742, 35.757, 27.661	72, 62, 62	Rutin	5280805	−7.66
CASP3	1GFW	36.742, 35.757, 27.661	72, 62, 62	Rosmarinic acid	5281792	−7.30
CASP3	1GFW	36.742, 35.757, 27.661	72, 62, 62	Salvianolic acid A	5281793	−8.24
CASP3	1GFW	36.742, 35.757, 27.661	72, 62, 62	Tanshinone IIA	164676	−6.76
TNF	2AZ5	−9.511, 68.418, 17.261	50, 44, 40	Caffeic acid	689043	−4.93
TNF	2AZ5	−9.511, 68.418, 17.261	50, 44, 40	Rutin	5280805	−7.13
TNF	2AZ5	−9.511, 68.418, 17.261	50, 44, 40	Cryptotanshinone	160254	−7.89
TP53	6GGA	88.807, 90.005, −45.360	48, 56, 34	Tanshinone IIA	164676	−8.73
BCL2	4IEH	12.626, 26.794, 11.105	60, 40, 32	Salvianolic acid A	5281793	−5.92
BCL2	4IEH	12.626, 26.794, 11.105	60, 40, 32	Tanshinone IIA	164676	−8.10
BCL2L1	3ZLN	−16.654, −5.777, 11.777	28, 46, 48	Cryptotanshinone	160254	−8.25
FOS	1A02	42.546, 35.426, 54.717	40, 54, 70	Tanshinone IIA	164676	−8.43

### 3.5 Experimental validation

#### 3.5.1 Effect of Danhong injection on apoptotic markers in rat heart tissue

**FIGURE 6:**
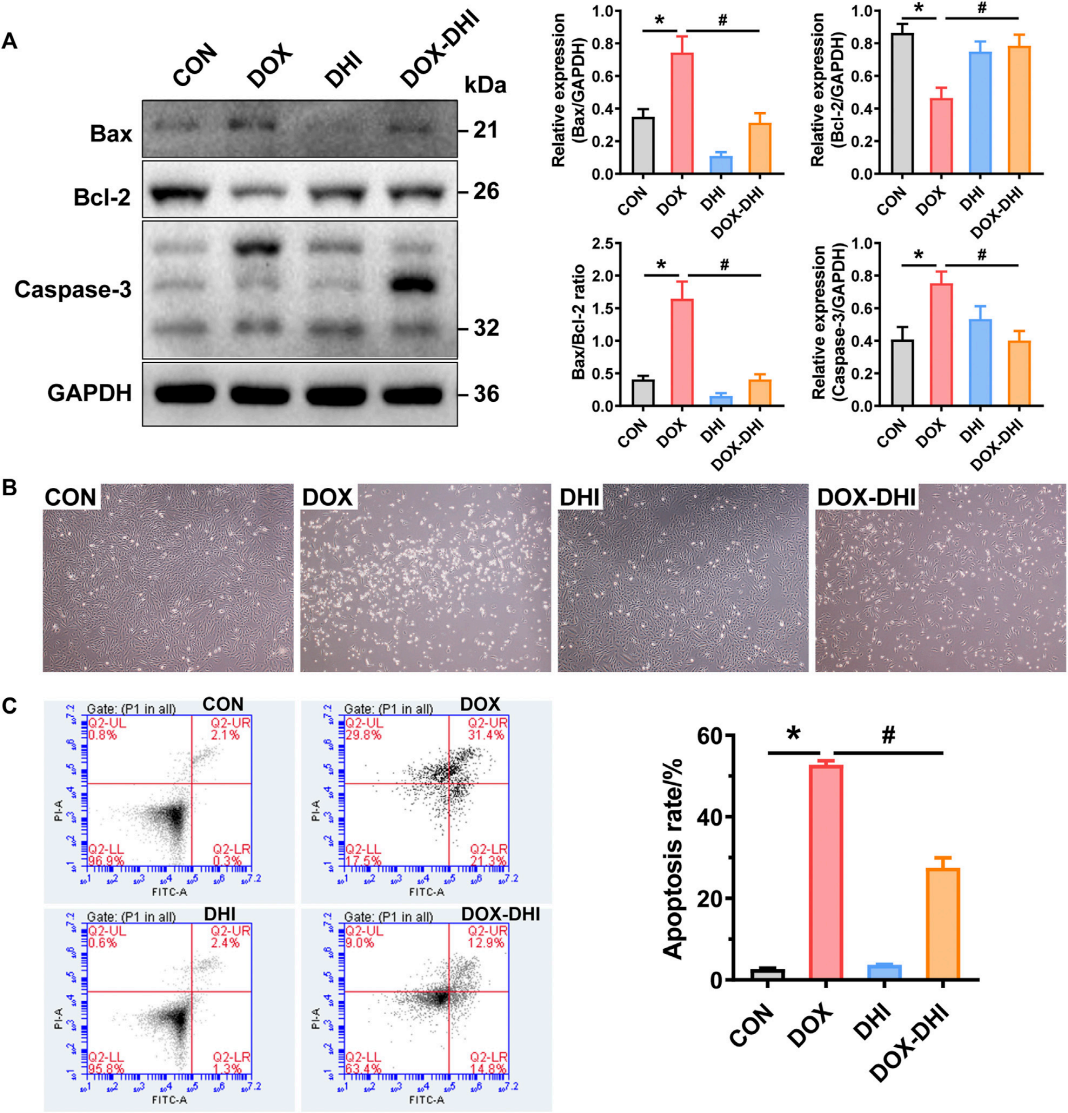
DHI attenuates DOX-induced cardiotoxicity by regulating the apoptosis pathway. **(A)** Detection of the expressions of cardiac apoptotic markers (Bax, Bcl-2, and caspase-3) in rat heart tissue by Western blot analysis. **(B)** Cell morphology of H9c2 cells after treatment with DOX and DHI. **(C)** Measurement of cell apoptosis in H9c2 cells by flow cytometry assay. CON: normal control; DHI: Danhong injection; DOX: doxorubicin. Data are expressed as mean ± SEM (*n* = 3). ^*^ *p* < 0.05 versus the CON group. ^#^ *p* < 0.05 versus the DOX group.

To experimentally validate the effect of DHI on DOX-induced apoptosis, the apoptotic markers Bax, Bcl-2, and caspase-3 in rat heart tissue were first assessed using western blot analysis (Figure 6A). The results showed that the expression of pro-apoptotic protein Bax was increased, while the anti-apoptotic protein Bcl-2 was downregulated in DOX-exposed animals when compared to the control group (*p* < 0.05). Treatment with DHI eliminated this trend (*p* < 0.05) and attenuated the DOX-induced increase in the Bax/Bcl-2 ratio. Moreover, the elevated level of caspase-3 was also observed in DOX-intoxicated rats and was reduced by the DHI treatment (*p* < 0.05). These results suggested that DHI attenuated DOX-induced myocardial injury via anti-apoptotic effect.

#### 3.5.2 Effect of Danhong injection on apoptosis in H9c2 cells

To further confirm the anti-apoptotic effect of DHI, a DOX-induced H9c2 cell model was introduced in the present study. After treatment with DOX and DHI for 48 h, the cell morphology was observed under a microscope at ×40 magnification (Figure 6B). Cells grew well and adhered firmly both in the control and DHI groups. While DOX administration caused dramatical changes in cell morphology and reduced cell attachment, with an increasing number of floating cells. The cell morphology in the DOX–DHI group was improved when compared to DOX group, but there were still some floating cells.

The effect of DHI on DOX-induced apoptosis rate was measured by flow cytometry with Annexin V-FITC/PI double staining. As shown in Figure 6C, the apoptosis rate of the DOX group was significantly increased when compared to the control group (*p* < 0.05), whereas a significant decreasing trend was observed in the cells receiving both DOX and DHI (*p* < 0.05). Moreover, DHI treatment had little effect on cell apoptosis (*p* > 0.05). Thus, these findings further demonstrate that DHI plays an essential role in DOX-induced cardiotoxicity by regulating cell apoptosis.

## 4 Discussion

DOX-induced cardiotoxicity is currently a conundrum in tumor chemotherapy. It is of great significance to find a safe and effective therapeutic strategy to reduce cardiac toxicity. DHI is derived from two common medicinal herbs *Salvia miltiorrhiza* Bunge [Lamiaceae] and *Carthamus tinctorius* L. [Asteraceae]. It is one of the most widely prescribed Chinese medicine injections. Our previous study has reported the protective effect of DHI on DOX-induced cardiotoxicity *in vitro* (Yi et al., 2018). However, the effect and mechanisms have yet to be deciphered *in vivo*. Thus, the present study was conducted to investigate the protective effect of DHI on DOX-induced cardiotoxicity and its potential molecular mechanisms by integrating chemical profiling, pharmacodynamics, network pharmacology, molecular docking, and experimental validation.

To evaluate the potential effect of DHI on attenuating DOX-induced cardiotoxicity, a chronic toxicity rat model was employed. The results showed that DOX administration led to a significant reduction in BW, HW, and HI. The ECG and histopathological examination results revealed the myocardial dysfunction and cardiac tissue damage induced by DOX. Meanwhile, serum ALT, AST, LDH, CK, and CK-MB levels were significantly elevated in DOX-intoxicated rats. Cardiac oxidative stress markers SOD and GSH-Px levels decreased, while MDA level was increased in the DOX group. These results demonstrate that administration of DOX caused evident cardiotoxicity, which is consistent with the findings in other studies (Sahu et al., 2016; Ye et al., 2020; Eisvand et al., 2022). In addition, DHI treatment in DOX-exposed animals significantly eliminated the BW, HW, and ECG abnormalities, caused a reduction in serum biochemical indicators levels, and restored the activities of cardiac oxidative enzymes. These data suggested that DHI exerted a cardioprotective effect against DOX-induced cardiotoxicity.

Many studies have demonstrated that induced cardiotoxicity occurs at a cumulative DOX dosage of 15 mg/kg in SD rats (Fouad et al., 2013; Tang et al., 2013; Chen et al., 2014; Ma et al., 2016). The present study showed that six equal injections of DOX (2.5 mg/kg each) over a 2-week period led to a BW and HW loss, which was in accordance with the results by other studies (Mantawy et al., 2014; Eisvand et al., 2022). It has been suggested that gastrointestinal side effects induced by DOX caused a reduction in the food intake of rats, which in turn affected the increase in BW and HW (Sahu et al., 2016; Zare et al., 2019). It has also been demonstrated that a decrease in HW was due to cytoplasmic vacuolization and myofibril loss, which was attributed to the dilation or distention of T-tubules and sarcoplasmic reticulum in cardiomyocytes (Carvalho et al., 2014; Zare et al., 2019). The present study demonstrated similar findings, where the DHI treatment prevented the loss of BW and HW. It is possible that DHI can exert a cardioprotective effect by improving cytoplasmic vacuolization and myofibril loss. However, histopathological examination of cardiac tissues showed no obvious improvements when used in combination with DHI, which may be related to the DHI dosage and duration. The ECG performance in DOX-intoxicated rats showed bradycardia, deepening S-wave, high amplitude T-wave, and prolongation of PR, QT, and QTc intervals. These alterations reflected cardiac dysrhythmias, conduction disturbances, and left-ventricular function attenuation (Mantawy et al., 2014). Analogous ECG changes were discovered in previous studies (Elberry et al., 2010; Saeed et al., 2015). However, the application of DHI restored the ECG to normal.

It has also been suggested that a deficiency in oxygen or nutrients in the myocardium may lead to cardiac cell membrane damage and further result in the release of myocardial enzymes into the serum. Therefore, the serum levels of myocardial enzymes, such as ALT, AST, LDH, CK, and CK-MB, are considered to be indicators of cardiac injury (Yu et al., 2017). The present data showed that the serum levels of ALT, AST, LDH, CK, and CK-MB were significantly increased in the presence of DOX, while DHI prevented the elevation of these enzyme levels. Thus, DHI reduced the increase in serum cardiac injury-related markers and alleviated DOX-induced myocardial damage by maintaining the structural integrity of cardiomyocyte membrane. Furthermore, growing evidence suggests that oxidative stress is associated with the pathogenesis of DOX-induced cardiotoxicity (Yu et al., 2018; Yu et al., 2020; Chan et al., 2021). DOX preferentially accumulates in cardiac mitochondria and disrupts the electron transport chain, which results in a substantial production of free radicals. Under normal physiological conditions, the endogenous defense system, including antioxidant enzymes SOD and GSH-Px, can eliminate free radicals, inhibit lipid peroxidation, and further prevent cardiomyocyte damage. In DOX-exposed animals, excess-free radicals lead to a consumption of antioxidant enzymes and ultimately cause myocardial damage (Alpsoy et al., 2013; Zare et al., 2019). The present study showed that SOD and GSH-Px levels decreased, while the indicator of lipid peroxidation MDA level was increased in DOX-intoxicated rats. This meant that DOX induced cardiotoxicity by increasing oxidative damage, which finally manifested in the heart tissue. Treatment with DHI prevented the increase in MDA level and restored the activities of cardiac oxidative enzymes.

To further uncover the molecular mechanisms of DHI against DOX-induced cardiotoxicity, network pharmacology analysis was performed to predict active compounds, potential targets, and key pathways. The present study collected chemical composition data from HPLC−ESI-Q-TOF-MS/MS analysis and acquired target information from the online databases (*e.g.*, TCMSP, SwissTargetPrediction, PharmGKB, and GeneCards), and then assembled an active compound-potential target network to explore the cardioprotective mechanisms. The network consisted of 14 active compounds, 15 potential targets, and 30 edges. Of these, the 15 potential targets were obtained by overlapping the targets related to DHI and DOX-induced cardiotoxicity, including SOD1, TIMP1, NOS3, TNF, MMP1, MMP2, NOS2, TOP2A, CASP3, XDH, BCL2, BCL2L1, FOS, TP53, and ECE1, which were considered as the potential targets of DHI against DOX-induced cardiotoxicity. In addition, 14 active compounds were connected to the 15 potential targets, such as danshensu, salvianolic acid A, salvianolic acid B, rosmarinic acid, and tanshinone IIA. Previous studies have demonstrated that salvianolic acid B, a major bioactive component in DHI with a promising cardiovascular protective effect, protected against DOX-induced cardiac dysfunction by inhibiting endoplasmic reticulum stress-mediated cardiomyocyte apoptosis (Wang et al., 2011; Chen et al., 2016; Chen et al., 2017). Other studies have suggested that danshensu, salvianolic acid A, rosmarinic acid, and tanshinone IIA also have significant effects in treatment or prevention of DOX-induced cardiotoxicity (Wang et al., 2016; Wang et al., 2019; Zhang et al., 2019; Wu et al., 2021). These results indicate that these active compounds may be the key substances of DHI in exerting its cardioprotective effects.

KEGG pathway analysis showed that the overlapping targets were mainly enriched in atherosclerosis, apoptosis, AGE-RAGE signaling pathway, and other disease-related pathways. In particular, six targets were highly enriched in the apoptosis pathway, including CASP3, BCL2, TP53, TNF, BCL2L1, and FOS. The results of molecular docking implied that the active compounds in DHI (rutin, rosmarinic acid, salvianolic acid A, tanshinone IIA, caffeic acid, and cryptotanshinone) had good binding affinities with the corresponding apoptosis pathway-related targets, which indicated the therapeutic effect of DHI was probably associated with the apoptosis pathway. Recently, the role of cardiomyocyte apoptosis in DOX-induced cardiotoxicity that may be mediated by the overproduction of free radicals has attracted increasing attention. DOX-induced oxidative stress activates the intrinsic and extrinsic mitochondrial-related apoptosis pathways, and ultimately leads to cardiomyocyte loss in the myocardium (Kluza et al., 2004; Zare et al., 2019). The present study shows that DOX increased the MDA level and inhibited the activities of SOD and GSH-Px, which meant the excessive accumulation of ROS. The evoked oxidative stress further stimulates the heat shock factor 1 (HSF-1), activates p53 to generate pro-apoptotic factors, and leads to cardiac cell apoptosis (Vedam et al., 2010; Rawat et al., 2021). It is well-documented that the levels of pro-apoptotic proteins, such as Bax, are up-regulated and anti-apoptotic proteins, such as Bcl-2, are down-regulated during apoptosis (Sahu et al., 2016; Azizi et al., 2017). This process induces mitochondrial permeability transition and leads to the release of caspase-3 from the mitochondria to the cytosol (Crompton, 2000; Mantawy et al., 2014). Western blot analysis of rat heart tissue revealed that DHI treatment significantly increased the level of Bcl-2 expression and prevented the elevation of Bax and caspase-3 expression. *In vitro* experiment further confirmed that DHI improved the cell morphology and significantly inhibited the apoptosis induced by DOX. These findings are consistent with the network pharmacology results and suggest that DHI exerted a therapeutic effect on DOX-induced cardiotoxicity via the apoptosis pathway.

## 5 Conclusion

In summary, LC−MS/MS analysis of DHI identified 56 compounds, mainly belonging to phenolic acids, tanshinones, and flavonoids. In DOX-intoxicated rats, DHI treatment prevented the reduction in BW and HW, improved ECG abnormalities, reduced the serum levels of myocardial injury markers, and restored the activities of cardiac oxidative enzymes. Network pharmacology-based strategy demonstrated that DHI played an essential role in DOX-induced cardiotoxicity through multiple compounds, targets, and pathways. Molecular docking confirmed that the active compounds in DHI had good binding activities with the apoptosis targets. Further *in vivo* and *in vitro* experiments showed that DHI exerted the cardioprotective effect primarily by regulating the apoptosis pathway. Thus, this study can serve as a reference for the novel application of DHI and provides a new potential treatment option for clinical DOX-induced myocardial damage.

## Data Availability

The original contributions presented in the study are included in the article/Supplementary Material; further inquiries can be directed to the corresponding authors.
