# Supplementary material for: Danhong injection attenuates doxorubicin-induced cardiotoxicity in rats via suppression of apoptosis: network pharmacology analysis and experimental validation
Source: Front Pharmacol. 2022 Aug 22;13:929302. doi: 10.3389/fphar.2022.929302 (PMC9441549; doi:10.3389/fphar.2022.929302)
Supplement: Supplementary file 1 [file DataSheet1.docx]

Supplementary Material

***Contents Page***

**Supplementary Figures**

**Supplementary Figure S1** Chemical structures of the constituents identified in DHI 2&3

**Supplementary Figure S2** The TCMP-compound-target-disease network 4

**Supplementary Figure S3** The top 10 enriched GO terms of the potential targets 5

**Supplementary Tables**

**Supplementary Table S1** Identification of chemical constituents in DHI by HPLC–ESI-Q-TOF-MS/MS 6

**Supplementary Table S2** The detailed information of candidate active compounds in DHI 12

**Supplementary Table S3** The detailed information of the potential targets of DHI against DOX-induced cardiotoxicity 13





**Supplementary Figure S1.** Chemical structures of the constituents identified in DHI.





**Supplementary Figure S1.** *(continued)*


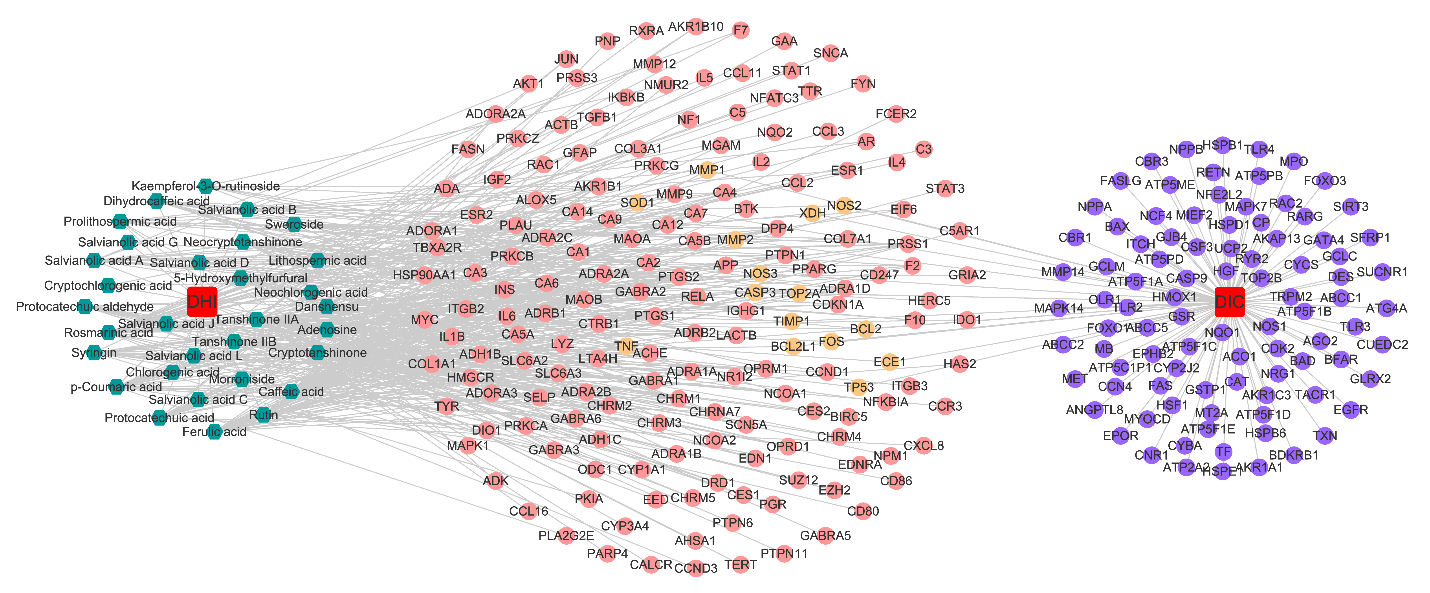


**Supplementary Figure S2.** The TCMP-compound-target-disease network. The green hexagons represent the candidate active components in DHI, the pink circles represent the targets of candidate active components, the purple circles represent the targets related to DOX-induced cardiotoxicity, and the yellow circles represent the overlapping targets between compound targets and disease targets.


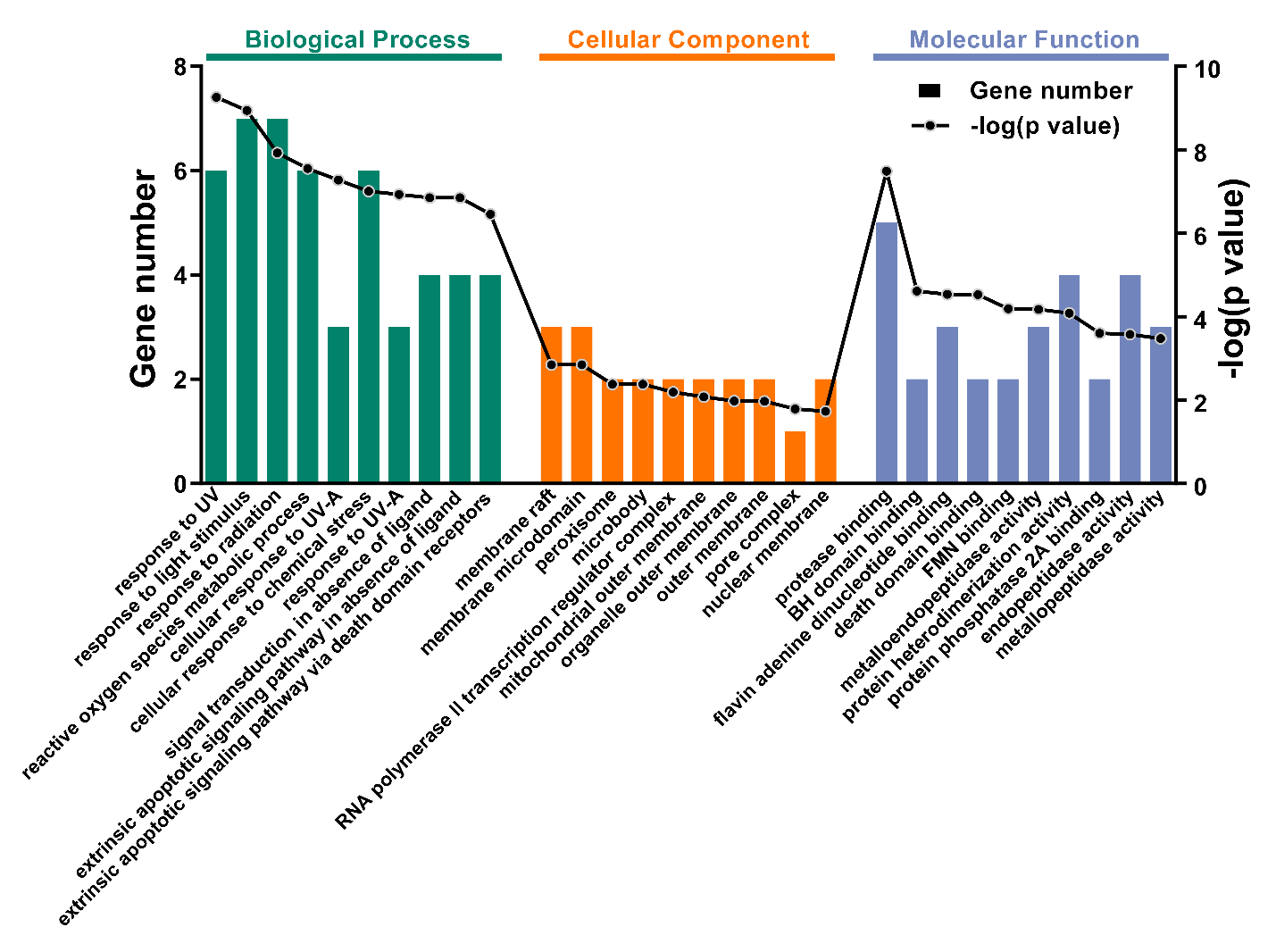


**Supplementary Figure S3.** The top 10 enriched GO terms of the potential targets.

**Supplementary Table S1.** Identification of chemical constituents in DHI by HPLC–ESI-Q-TOF-MS/MS.

| No. | t_R_ (min) | Identification | Formula | Selected ion | Measured *m/z* | Calculated *m/z* | Error (ppm) | Fragment ions (*m/z*) | Source |
| --- | --- | --- | --- | --- | --- | --- | --- | --- | --- |
| 1^a^ | 5.677 | Uridine | C_9_H_12_N_2_O_6_ | [M−H]^−^ | 243.0616 | 243.0623 | −2.6 | 243.0618 (17) [M−H]^−^,  200.0567 (32) [M−H−HNCO]^−^,  110.0263 (100) [M−H−rib]^−^ | F |
|  |  |  |  | [M+H]^+^ | 245.0766 | 245.0768 | −0.9 | 113.0351 (100) [M+H−rib]^+^ |  |
| 2 | 5.866 | Adenosine | C_10_H_13_N_5_O_4_ | [M+H]^+^ | 268.1040 | 268.1040 | 0.0 | 136.0622 (100) [M+H−rib]^+^,  119.0356 (9) [M+H−rib−NH_3_]^+^ | F |
| 3 | 7.336 | Guanosine | C_10_H_13_N_5_O_5_ | [M−H]^−^ | 282.0832 | 282.0844 | −4.2 | 282.0853 (21) [M−H]^−^,  150.0429 (100) [M−H−rib]^−^,  133.0162 (42) [M−H−rib−NH_3_]^−^,  108.0214 (12) [M−H−rib−HNCO]^−^ | F |
|  |  |  |  | [M+H]^+^ | 284.0991 | 284.0990 | 0.7 |  |  |
| 4 | 9.847 | p-Hydroxybenzoic acid-*O*-glucoside | C_13_H_16_O_8_ | [M−H]^−^ | 299.0763 | 299.0772 | −3.0 | 137.0247 (100) [M−H−glu]^−^ | S/F |
|  |  |  | C_14_H_18_O_10_ | [M+COOH]^−^ | 345.0811 | 345.0827 | −4.7 |  |  |
| 5^a^ | 10.651 | 5-Hydroxymethylfurfural | C_6_H_6_O_3_ | [M+H]^+^ | 127.0391 | 127.0390 | 0.9 | 109.0287 (100) [M+H−H_2_O]^+^ | Byproduct |
| 6 | 11.365 | Phenylalanine | C_9_H_11_NO_2_ | [M−H]^−^ | 164.0719 | 164.0717 | 0.9 | 147.0455 (100) [M−H−NH_3_]^−^,  103.0569 (67) [M−H−NH_3_−CO_2_]^−^ | F |
|  |  |  |  | [M+H]^+^ | 166.0863 | 166.0863 | 0.3 |  |  |
| 7 | 12.115 | 2-Hydroxy-3',4'-dihydroxyacetophenone | C_8_H_8_O_4_ | [M−H]^−^ | 167.0350 | 167.0350 | 0.2 | 167.0342 (37) [M−H]^−^,  123.0453 (100) [M−H−CO_2_]^−^ | S/F |
| 8 | 12.118 | Not identified | C_32_H_30_O_10_ | [M+H]^+^ | 575.1880 | 575.1912 | −5.4 | 575.1901 (26) [M+H]^+^,  413.1358 (100) [M+H−glu]^+^,  307.0935 (28) [M+H−glu−C_6_H_2_O_2_]^+^ | S/F |
| 9^a^ | 12.456 | Danshensu | C_9_H_10_O_5_ | [M−H]^−^ | 197.0456 | 197.0456 | 0.1 | 197.0458 (7) [M−H]^−^,  179.0352 (23) [M−H−H_2_O]^−^,  135.0460 (100) [M−H−H_2_O−CO_2_]^−^,  123.0466 (74) [M−H−CH_2_O−CO_2_]^−^ | S |
|  |  |  | C_18_H_20_O_10_ | [2M−H]^−^ | 395.0968 | 395.0984 | −4.0 |  |  |
| 10^a^ | 13.876 | Protocatechuic acid | C_7_H_6_O_4_ | [M−H]^−^ | 153.0192 | 153.0193 | −0.9 | 109.0288 (100) [M−H−CO_2_]^−^ | S |
| 11 | 16.084 | Neochlorogenic acid | C_16_H_18_O_9_ | [M−H]^−^ | 353.0861 | 353.0878 | −4.8 | 353.0894 (11) [M−H]^−^,  191.0567 (100) [M−H−caffeoyl]^−^,  179.0353 (44) [CA−H]^−^,  135.0460 (28) [CA−H−CO_2_]^−^ | F |
|  |  |  | C_32_H_36_O_18_ | [2M−H]^−^ | 707.1808 | 707.1829 | −3.0 |  |  |
| 12 | 16.973 | Dihydrocaffeic acid | C_9_H_10_O_4_ | [M−H]^−^ | 181.0508 | 181.0506 | 1.0 | 181.0503 (23) [M−H]^−^,  163.0392 (46) [M−H−H_2_O]^−^,  135.0453 (100) [M−3H−CO_2_]^−^,  119.0504 (45) [M−H−H_2_O−CO_2_]^−^ | S |
| 13^a^ | 18.865 | Protocatechuic aldehyde | C_7_H_6_O_3_ | [M−H]^−^ | 137.0243 | 137.0244 | −0.9 | 137.0229 (100) [M−H]^−^,  136.0144 (59) [M−2H]^−^,  108.0205 (44) [M−H−CHO]^−^ | S |
| 14 | 19.339 | Isopropylmalic acid | C_7_H_12_O_5_ | [M−H]^−^ | 175.0614 | 175.0612 | 1.0 | 175.0617 (28) [M−H]^−^,  157.0515 (11) [M−H−H_2_O]^−^,  131.0714 (9) [M−H−CO_2_]^−^,  115.0415 (100) [M−2H−CH_3_CO_2_]^−^,  113.0616 (43) [M−H−H_2_O−CO_2_]^−^ | F |
| 15 | 20.066 | Caffeic acid-*O*-hexoside | C_15_H_18_O_9_ | [M−H]^−^ | 341.0863 | 341.0878 | −4.3 | 341.0884 (13) [M−H]^−^,  179.0349 (100) [M−H−glu]^−^,  135.0458 (65) [M−H−glu−CO_2_]^−^ | F |
| 16 | 20.184 | Syringin | C_17_H_24_O_9_ | [M+COOH]^−^ | 417.1391 | 417.1402 | −2.6 | 417.1780 (38) [M+COOH]^−^,  209.0815 (100) [M−H−glu]^−^,  194.0577 (46) [M−H−glu−CH_3_]^−^ | S |
| 17 | 20.637 | Chlorogenic acid | C_16_H_18_O_9_ | [M−H]^−^ | 353.0860 | 353.0878 | −5.1 | 191.0564 (100) [M−H−caffeoyl]^−^ | F |
|  |  |  | C_32_H_36_O_18_ | [2M−H]^−^ | 707.1793 | 707.1829 | −5.1 |  |  |
| 18 | 20.733 | Coumaric acid-*O*-hexoside | C_15_H_18_O_8_ | [M−H]^−^ | 325.0918 | 325.0929 | −3.4 | 163.0405 (63) [M−H−glu]^−^,  119.0514 (100) [M−H−glu−CO_2_]^−^ | F |
|  |  |  | C_15_H_19_ClO_8_ | [M+Cl]^−^ | 361.0678 | 361.0696 | −4.9 | 361.0692 (25) [M+Cl]^−^,  163.0398 (100) [M−H−glu]^−^,  119.0512 (72) [M−H−glu−CO_2_]^−^ |  |
| 19 | 20.875 | 6-Hydroxykaempferol-di-*O*-glucoside | C_27_H_30_O_17_ | [M+H]^+^ | 627.1563 | 627.1556 | 1.1 | 465.1035 (15) [M+H−glu]^+^,  303.0507 (100) [M+H−2glu]^+^ | F |
| 20 | 21.218 | 6-Hydrokaempferol-di-*O*-glucoside | C_27_H_30_O_17_ | [M+H]^+^ | 627.1571 | 627.1556 | 2.4 | 465.1034 (9) [M+H−glu]^+^,  303.0512 (100) [M+H−2glu]^+^ | F |
| 21 | 21.224 | Hydroxysafflor yellow A | C_27_H_32_O_16_ | [M−H]^−^ | 611.1593 | 611.1618 | −4.1 | 611.1685 (100) [M−H]^−^,  491.1234 (36) [M−H−C_4_H_8_O_4_]^−^,  473.1129 (9) [M−H−C_4_H_8_O_4_−H_2_O]^−^,  403.1060 (19) [M−H−C_7_H_12_O_7_]^−^,  325.0735 (20) [M−H−C_9_H_18_O_10_]^−^,  283.0627 (10) [M−C_4_H_8_O_4_−C_4_H_8_O_4_−CO−H_2_O−C_2_H_2_O]^−^ | F |
| 22 | 21.379 | Cryptochlorogenic acid | C_16_H_18_O_9_ | [M−H]^−^ | 353.0863 | 353.0878 | −4.2 | 353.0899 (19) [M−H]^−^,  191.0565 (95) [M−H−caffeoyl]^−^,  179.0353 (81) [CA−H]^−^,  173.0459 (100) [M−H−caffeoyl−H_2_O]^−^,  135.0456 (71) [CA−H−CO_2_]^−^ | F |
|  |  |  | C_32_H_36_O_18_ | [2M−H]^−^ | 707.1814 | 707.1829 | −2.1 |  |  |
| 23 | 21.593 | Ferulic acid | C_10_H_10_O_4_ | [M−H]^−^ | 193.0504 | 193.0506 | −1.0 | 149.0614 (100) [M−H−CO_2_]^−^,  134.0380 (12) [M−H−CO_2_−CH_3_]^−^ | S |
| 24 | 22.589 | Caffeic acid | C_9_H_8_O_4_ | [M−H]^−^ | 179.0348 | 179.0350 | −0.8 | 179.0356 (6) [M−H]^−^,  135.0460 (100) [M−H−CO_2_]^−^,  134.0377 (44) [M−2H−CO_2_]^−^ | S |
| 25 | 22.837 | Roseoside | C_19_H_30_O_8_ | [M+COOH]^−^ | 431.1905 | 431.1923 | −4.1 | 431.1951 (45) [M+COOH]^−^,  385.1892 (100) [M−H]^−^,  223.1337 (34) [M−H−glu]^−^,  205.1233 (24) [M−H−glu−H_2_O]^−^ | F |
| 26 | 22.891 | Sweroside | C_16_H_22_O_9_ | [M−H]^−^ | 357.1175 | 357.1191 | −4.4 | 195.0665 (100) [M−H−glu]^−^ | F |
| 27 | 23.024 | Morroniside | C_17_H_26_O_11_ | [M−H]^−^ | 405.1402 | 405.1402 | −0.1 | 405.1758 (38) [M−H]^−^,  243.1721 (100) [M−H−glu]^−^ | F |
| 28 | 26.021 | Coumaric acid-*O*-hexoside isomer | C_15_H_18_O_8_ | [M−H]^−^ | 325.0917 | 325.0929 | −3.6 | 119.0520 (100) [M−H−glu−CO_2_]^−^ | F |
| 29 | 26.534 | Prolithospermic acid | C_18_H_14_O_8_ | [M−H]^−^ | 357.0602 | 357.0616 | −3.9 | 357.0621 (27) [M−H]^−^,  313.0732 (35) [M−H−CO_2_]^−^,  269.0823 (100) [M−H−2CO_2_]^−^,  203.0351 (40) [M−H−CO_2_−C_6_H_6_O_2_]^−^,  159.0455 (41) [M−H−2CO_2_−C_6_H_6_O_2_]^−^,  109.0307 (61) [C_6_H_6_O_2_−H]^−^ | S |
| 30 | 26.770 | Carthamidin-di-*O*-glucoside | C_20_H_36_O_21_ | [M−H]^−^ | 611.1585 | 611.1618 | −5.3 | 611.1674 (84) [M−H]^−^,  449.1115 (100) [M−H−glu]^−^,  287.0563 (52) [M−H−2glu]^−^ | F |
| 31^a^ | 27.391 | p-Coumaric acid | C_9_H_8_O_3_ | [M−H]^−^ | 163.0402 | 163.0401 | 1.0 | 119.0514 (100) [M−H−CO_2_]^−^ | F |
| 32 | 28.284 | Rutin | C_27_H_30_O_16_ | [M+H]^+^ | 611.1627 | 611.1607 | 3.3 | 303.0509 (100) [M+H−rutin]^+^ | F |
| 33 | 29.729 | Salvianolic acid I/J | C_27_H_22_O_12_ | [M−H]^−^ | 537.1019 | 537.1039 | −3.6 | 537.1082 (5) [M−H]^−^,  493.1176 (9) [M−H−CO_2_]^−^,  313.0728 (10) [M−H−CO_2_−CA]^−^,  295.0616 (100) [M−H−CO_2_−DSS]^−^,  185.0246 (69) [M−H−CO_2_−DSS−C_6_H_6_O_2_]^−^,  109.0311 (14) [C_6_H_6_O_2_−H]^−^ | S |
| 34 | 30.753 | Salvianolic acid I/J | C_27_H_22_O_12_ | [M−H]^−^ | 537.1018 | 537.1039 | −3.8 | 537.1086 (6) [M−H]^−^,  493.1176 (10) [M−H−CO_2_]^−^,  313.0727 (10) [M−H−CO_2_−CA]^−^,  295.0618 (100) [M−H−CO_2_−DSS]^−^,  185.0248 (64) [M−H−CO_2_−DSS−C_6_H_6_O_2_]^−^,  109.0315 (12) [C_6_H_6_O_2_−H]^−^ | S |
| 35 | 31.227 | Salviaflaside | C_24_H_26_O_13_ | [M−H]^−^ | 521.1280 | 521.1301 | −3.9 | 521.1346 (27) [M−H]^−^,  359.0795 (89) [M−H−glu]^−^,  323.0790 (100) [M−H−DSS]^−^,  197.0463 (28) [DSS−H]^−^,  179.0355 (29) [DSS−H−H_2_O]^−^,  161.0255 (78) [M−H−glu−DSS]^−^ | S |
| 36 | 31.627 | Kaempferol-3-*O*-rutinoside | C_27_H_30_O_15_ | [M−H]^−^ | 593.1500 | 593.1512 | −2.1 | 593.1575 (100) [M−H]^−^,  285.0415 (78) [M−H−rutin]^−^ | F |
| 37^a^ | 33.898 | Salvianolic acid D | C_20_H_18_O_10_ | [M−H]^−^ | 417.0813 | 417.0827 | −3.5 | 417.0849 (11) [M−H]^−^,  197.0462 (60) [DSS−H]^−^,  179.0355 (40) [DSS−H−H_2_O]^−^,  175.0405 (100) [M−H−CO_2_−DSS]^−^,  135.0461 (16) [DSS−H−H_2_O−CO_2_]^−^ | S |
| 38 | 34.644 | Salvianolic acid G | C_18_H_12_O_7_ | [M−H]^−^ | 339.0499 | 339.0510 | −3.4 | 339.0530 (53) [M−H]^−^,  321.0418 (23) [M−H−H_2_O]^−^,  295.0623 (45) [M−H−CO_2_]^−^,  280.0384 (100) [M−H−C_2_H_3_O_2_]^−^,  252.0430 (49) [M−H−CO_2_−C_2_H_3_O]^−^,  224.0476 (18) [M−H−CO2−C_2_H_3_O−CO]^−^ | S |
| 39 | 36.224 | Salvianolic acid F | C_17_H_14_O_6_ | [M−H]^−^ | 313.0705 | 313.0718 | −4.1 | 313.0729 (32) [M−H]^−^,  269.0822 (31) [M−H−CO_2_]^−^,  159.0452 (30) [M−H−CO_2_−C_6_H_6_O_2_]^−^,  109.0305 (100) [C_6_H_6_O_2_−H]^−^ | S |
| 40 | 36.753 | Salvianolic acid E | C_36_H_30_O_16_ | [M−H]^−^ | 717.1457 | 717.1461 | −0.6 | 717.1552 (14) [M−H]^−^,  519.0969 (100) [M−H−DSS]^−^,  339.0516 (45) [M−H−DSS−CA]^−^,  321.0409 (97) [M−H−2DSS]^−^,  295.0618 (21) [M−H−DSS−CA−CO_2_]^−^ | S |
| 41^a^ | 37.731 | Rosmarinic acid | C_18_H_16_O_8_ | [M−H]^−^ | 359.0759 | 359.0772 | −3.6 | 197.0454 (42) [M−H−caffeoyl]^−^,  179.0347 (37) [M−H−CA]^−^,  161.0245 (100) [M−H−DSS]^−^,  135.0452 (22) [M−H−CA−CO_2_]^−^,  133.0298 (21) [M−H−DSS−CO]^−^ | S |
|  |  |  | C_36_H_32_O_16_ | [2M−H]^−^ | 719.1616 | 719.1618 | −0.2 |  |  |
| 42 | 39.067 | Isomer of salvianolic acid A | C_26_H_22_O_10_ | [M−H]^−^ | 493.1124 | 493.1140 | −3.3 | 493.1173 (14) [M−H]^−^,  295.0619 (100) [M−H−DSS]^−^,  277.0507 (4) [M−H−DSS−H_2_O]^−^,  185.0243 (46) [M−H−DSS−C_6_H_6_O_2_]^−^,  109.0302 (15) [C_6_H_6_O_2_−H]^−^ | S |
| 43^a^ | 39.073 | Lithospermic acid | C_27_H_22_O_12_ | [M−H]^−^ | 537.1024 | 537.1039 | −2.7 | 493.1183 (12) [M−H−CO_2_]^−^,  295.0623 (100) [M−H−CO_2_−DSS]^−^,  185.0249 (60) [M−H−CO_2_−DSS−C_6_H_6_O_2_]^−^,  109.0304 (11) [C_6_H_6_O_2_−H]^−^ | S |
| 44 | 40.815 | 9''-Methyl lithospermate B | C_37_H_32_O_16_ | [M−H]^−^ | 731.1620 | 731.1618 | 0.3 | 731.1727 (12) [M−H]^−^,  551.1241 (7) [M−H−CA]^−^,  533.1138 (100) [M−H−DSS]^−^,  353.0678 (40) [M−H−DSS−CA]^−^,  335.0572 (57) [M−H−2DSS]^−^,  309.0771 (12) [M−H−DSS−CA−CO_2_]^−^ | S |
| 45^a^ | 42.446 | Salvianolic acid B | C_36_H_30_O_16_ | [M−H]^−^ | 717.1458 | 717.1461 | −0.4 | 717.1549 (8) [M−H]^−^,  519.0969 (68) [M−H−DSS]^−^,  339.0519 (45) [M−H−DSS−CA]^−^,  321.0412 (100) [M−H−2DSS]^−^,  295.0620 (23) [M−H−DSS−CA−CO_2_]^−^ | S |
| 46 | 43.066 | Salvianolic acid L | C_36_H_30_O_16_ | [M−H]^−^ | 717.1467 | 717.1461 | 0.8 | 717.1541 (10) [M−H]^−^,  519.0955 (57) [M−H−DSS]^−^,  339.0501 (33) [M−H−DSS−CA]^−^,  321.0392 (100) [M−H−2DSS]^−^,  295.0595 (17) [M−H−DSS−CA−CO_2_]^−^ | S |
| 47 | 44.124 | Ethyl lithospermic acid | C_29_H_26_O_12_ | [M−H]^−^ | 565.1338 | 565.1352 | −2.4 | 565.1401 (19) [M−H]^−^,  367.0839 (29) [M−H−DSS]^−^,  321.0409 (100) [M−H−DSS−CO_2_]^−^ | S |
| 48 | 44.929 | Dimethyl lithospermic acid | C_29_H_26_O_12_ | [M−H]^−^ | 565.1335 | 565.1352 | −2.9 | 565.1409 (30) [M−H]^−^,  367.0845 (36) [M−H−DSS]^−^,  321.0417 (100) [M−H−DSS−CO_2_]^−^ | S |
| 49^a^ | 45.979 | Salvianolic acid A | C_26_H_22_O_10_ | [M−H]^−^ | 493.1131 | 493.1140 | −1.9 | 493.1184 (8) [M−H]^−^,  313.0730 (11) [M−H−CA]^−^,  295.0618 (100) [M−H−DSS]^−^,  185.0246 (90) [M−H−DSS−C_6_H_6_O_2_]^−^,  109.0310 (22) [C_6_H_6_O_2_−H]^−^ | S |
|  |  |  | C_52_H_44_O_20_ | [2M−H]^−^ | 987.2361 | 987.2353 | 0.8 |  |  |
| 50 | 46.461 | Rosmarinic acid methyl ester | C_19_H_18_O_8_ | [M−H]^−^ | 373.0924 | 373.0929 | −1.3 | 179.0345 (11) [M−H−CA−CH_2_]^−^,  135.0456 (100) [M−H−CA−CO_2_−CH_2_]^−^ | S |
| 51 | 47.705 | 9'''-Methyl lithospermate B/isomer | C_37_H_32_O_16_ | [M−H]^−^ | 731.1620 | 731.1618 | 0.3 | 731.1715 (17) [M−H]^−^,  533.1144 (100) [M−H−DSS]^−^,  353.0689 (45) [M−H−DSS−CA]^−^,  335.0582 (58) [M−H−2DSS]^−^ | S |
| 52 | 48.722 | Salvianolic acid C | C_26_H_20_O_10_ | [M−H]^−^ | 491.0970 | 491.0984 | −2.8 | 491.1030 (49) [M−H]^−^,  311.0571 (75) [M−H−C_9_H_8_O_4_]^−^,  293.0465(100) [M−H−DSS]^−^,  249.0562 (32) [M−H−C_9_H_8_O_4_−CO_2_−H_2_O]^−^ | S |
| 53 | 49.356 | Tanshinone IIB | C_19_H_18_O_4_ | [M+H]^+^ | 311.1283 | 311.1278 | 1.8 | 311.1280 (30) [M+H]^+^,  265.1231 (64) [M+H−C_2_H_6_O]^+^,  221.1329 (85) [M+H−C_3_H_6_O_3_]^+^,  206.1095 (100) [M+H−C_4_H_9_O_3_]^+^,  191.0855 (88) [M+H−C_5_H_12_O_3_]^+^ | S |
| 54 | 50.179 | Neocryptotanshinone | C_19_H_22_O_4_ | [M+H]^+^ | 315.1593 | 315.1591 | 0.7 | 315.1604 (23) [M+H]^+^,  297.1493 (100) [M+H−H_2_O]^+^,  269.1544 (62) [M+H−H_2_O−CO]^+^,  253.1593 (49) [M+H−H_2_O−CO_2_]^+^,  251.1427 (41) [M+H−H_2_O−CH_2_O_2_]^+^ | S |
| 55 | 50.224 | Cryptotanshinone | C_19_H_20_O_3_ | [M+H]^+^ | 297.1490 | 297.1485 | 1.6 | 297.1502 (15) [M+H]^+^,  253.1595 (100) [M+H−CO_2_]^+^,  237.0913 (73) [M+H−C_2_H_4_O_2_]^+^,  223.1119 (72) [M+H−C_3_H_6_O_2_]^+^,  211.1112 (59) [M+H−C_4_H_6_O_2_]^+^,  165.0694 (44) [M+H−C_5_H_8_O_4_]^+^ | S |
| 56 | 50.565 | Tanshinone IIA | C_19_H_18_O_3_ | [M+H]^+^ | 295.1330 | 295.1329 | 0.3 | 295.2181 (86) [M+H]^+^,  277.1235 (86) [M+H−H_2_O]^+^,  251.1440 (86) [M+H−C_2_H_4_O]^+^,  191.0862 (100) [M+H−C_4_H_8_O_3_]^+^,  178.0774 (75) [M+H−C_5_H_9_O_3_]^+^ | S |

Abbreviation: Rib: ribose; Glu: glucosyl; Rutin: rutinosyl; DSS: danshensu; CA: caffeic acid; S: Radix Salviae Miltiorrhizae; F: Flos Carthami tinctorii.

^a^ Further confirmed with reference compounds.

**Supplementary Table S2.** The detailed information of candidate active compounds in DHI.

| ID | Compound | Formula | MW | CAS | DL |
| --- | --- | --- | --- | --- | --- |
| D1 | Adenosine | C_10_H_13_N_5_O_4_ | 267.28 | 5536-17-4 | 0.18 |
| D2 | Guanosine | C_10_H_13_N_5_O_5_ | 283.28 | 118-00-3 | 0.21 |
| D3 | p-Hydroxybenzoic acid-*O*-glucoside | C_13_H_16_O_8_ | 300.29 | N/A | 0.20 |
| D4 | 5-Hydroxymethylfurfural | C_6_H_6_O_3_ | 126.12 | 67-47-0 | 0.02 |
| D5 | Danshensu | C_9_H_10_O_5_ | 198.19 | 76822-21-4 | 0.06 |
| D6 | Protocatechuic acid | C_7_H_6_O_4_ | 154.13 | 99-50-3 | 0.04 |
| D7 | Neochlorogenic acid | C_16_H_18_O_9_ | 354.34 | 906-33-2 | 0.33 |
| D8 | Dihydrocaffeic acid | C_9_H_10_O_4_ | 182.19 | 71693-95-3 | 0.05 |
| D9 | Protocatechuic aldehyde | C_7_H_6_O_3_ | 138.13 | 139-85-5 | 0.03 |
| D10 | Syringin | C_17_H_24_O_9_ | 372.41 | 118-34-3 | 0.32 |
| D11 | Chlorogenic acid | C_16_H_18_O_9_ | 354.34 | 202650-88-2 | 0.33 |
| D12 | Hydroxysafflor yellow A | C_27_H_32_O_16_ | 612.59 | 78281-02-4 | 0.68 |
| D13 | Cryptochlorogenic acid | C_16_H_18_O_9_ | 354.34 | 905-99-7 | 0.33 |
| D14 | Ferulic acid | C_10_H_10_O_4_ | 194.2 | 537-98-4 | 0.06 |
| D15 | Caffeic acid | C_9_H_8_O_4_ | 180.17 | 501-16-6 | 0.05 |
| D16 | Roseoside | C_19_H_30_O_8_ | 386.49 | 54835-70-0 | 0.36 |
| D17 | Sweroside | C_16_H_22_O_9_ | 358.38 | 14215-86-2 | 0.38 |
| D18 | Morroniside | C_17_H_26_O_11_ | 406.43 | 25406-64-8 | 0.50 |
| D19 | Prolithospermic acid | C_18_H_14_O_8_ | 314.31 | N/A | 0.31 |
| D20 | p-Coumaric acid | C_9_H_8_O_3_ | 164.17 | 7400-08-0 | 0.04 |
| D21 | Rutin | C_27_H_30_O_16_ | 610.57 | 153-18-4 | 0.68 |
| D22 | Salvianolic acid I | C_27_H_22_O_12_ | 538.49 | 153765-45-8 | N/A |
| D23 | Salvianolic acid J | C_27_H_22_O_12_ | 538.49 | N/A | 0.72 |
| D24 | Salviaflaside | C_24_H_26_O_13_ | 522.46 | 178895-25-5 | N/A |
| D25 | Kaempferol-3-*O*-rutinoside | C_27_H_30_O_15_ | 594.57 | 17650-84-9 | 0.73 |
| D26 | Salvianolic acid D | C_20_H_18_O_10_ | 418.38 | 142998-47-8 | 0.50 |
| D27 | Salvianolic acid G | C_18_H_12_O_7_ | 340.3 | N/A | 0.61 |
| D28 | Salvianolic acid F | C_17_H_14_O_6_ | 314.29 | N/A | 0.26 |
| D29 | Salvianolic acid E | C_36_H_30_O_16_ | 718.66 | N/A | 0.39 |
| D30 | Rosmarinic acid | C_18_H_16_O_8_ | 360.34 | 20283-92-5 | 0.35 |
| D31 | Lithospermic acid | C_27_H_22_O_12_ | 538.49 | 28831-65-4 | 0.76 |
| D32 | 9''-Methyl lithospermate B | C_37_H_32_O_16_ | 732.69 | N/A | 0.39 |
| D33 | Salvianolic acid B | C_36_H_30_O_16_ | 718.66 | 115939-25-8 | 0.41 |
| D34 | Salvianolic acid L | C_36_H_30_O_16_ | 718.66 | N/A | 0.44 |
| D35 | Salvianolic acid A | C_26_H_22_O_10_ | 494.48 | 96574-01-5 | 0.70 |
| D36 | Salvianolic acid C | C_26_H_20_O_10_ | 492.46 | 115841-09-3 | 0.81 |
| D37 | Tanshinone IIB | C_19_H_18_O_4_ | 310.37 | 17397-93-2 | 0.45 |
| D38 | Neocryptotanshinone | C_19_H_22_O_4_ | 314.41 | 109664-02-0 | 0.32 |
| D39 | Cryptotanshinone | C_19_H_20_O_3_ | 296.39 | 35825-57-1 | 0.40 |
| D40 | Tanshinone IIA | C_19_H_18_O_3_ | 294.37 | 568-72-9 | 0.40 |

**Supplementary Table S3.** The detailed information of the potential targets of DHI against DOX-induced cardiotoxicity.

| No. | Gene Name | Gene Symbol | Uniprot ID |
| --- | --- | --- | --- |
| 1 | Superoxide Dismutase 1 | SOD1 | P00441 |
| 2 | TIMP Metallopeptidase Inhibitor 1 | TIMP1 | P01033 |
| 3 | Nitric Oxide Synthase 3 | NOS3 | P29474 |
| 4 | Tumor Necrosis Factor | TNF | P01375 |
| 5 | Matrix Metallopeptidase 1 | MMP1 | P03956 |
| 6 | Matrix Metallopeptidase 2 | MMP2 | P08253 |
| 7 | Nitric Oxide Synthase 2 | NOS2 | P35228 |
| 8 | DNA Topoisomerase II Alpha | TOP2A | P11388 |
| 9 | Caspase 3 | CASP3 | P42574 |
| 10 | Xanthine Dehydrogenase | XDH | P47989 |
| 11 | BCL2 Apoptosis Regulator | BCL2 | P10415 |
| 12 | BCL2 Like 1 | BCL2L1 | Q07817 |
| 13 | Fos Proto-Oncogene, AP-1 Transcription Factor Subunit | FOS | P01100 |
| 14 | Tumor Protein P53 | TP53 | P04637 |
| 15 | Endothelin Converting Enzyme 1 | ECE1 | P42892 |
